# β-Galactosidase expression on *Lactococcus* sp. KTH0-1S as a whole-cell biocatalyst for efficient lactosucrose production

**DOI:** 10.1371/journal.pone.0359091

**Published:** 2026-09-25

**Authors:** Nichapat Ariyakajorn, Napat Sunsern, Phitsanu Pinmanee, Boontiwa Ninchan, Pimsiriya Srila, Konlarat Phirom-on, Sivatch Imrattanarak, Wirat Vanichsriratana, Nisit Watthanasakphuban

**Affiliations:** 1 Department of Biotechnology, Faculty of Agro-Industry, Kasetsart University, Bangkok, Thailand; 2 Biocatalyst Technology Research Team, National Center of Genetic Engineering and Biotechnology (BIOTEC), Pathum Thani, Thailand; 3 Food Biotechnology Laboratory, Department of Biotechnology and Food Sciences, BOKU University, Vienna, Austria; Ruhr-Universitat Bochum, GERMANY

## Abstract

Lactosucrose is a functional trisaccharide with prebiotic properties and growing potential in food and agricultural applications; however, its industrial production is limited by low conversion efficiency and high downstream processing costs. In this study, a whole-cell biocatalyst was developed by displaying β-galactosidase on *Lactococcus* sp. KTH0-1S using a nisin-inducible expression system, the USP45 signal peptide, and a LysM anchoring domain. The engineered strain exhibited a maximum whole-cell β-galactosidase activity of 146.0 Miller units after 4 h of induction. The whole-cell enzyme efficiently catalyzed lactosucrose synthesis from lactose and sucrose, with optimal activity observed at pH 6.0 and 50 °C. Substrate molar ratio significantly influenced product formation, and the highest lactosucrose concentration (15,940 mg/L) was achieved at a lactose:sucrose ratio of 1:3. Reusability studies revealed high initial catalytic activity but limited stability upon repeated reaction cycles, likely due to the non-covalent nature of LysM-mediated surface anchoring. Molecular docking analysis supported the experimental observations by identifying a high-affinity substrate-binding pocket capable of accommodating both donor and acceptor substrates, providing a structural rationale for enhanced transgalactosylation under sucrose-enriched conditions. Overall, this study demonstrates the feasibility of using a whole-cell biocatalyst based on *Lactococcus* sp. for efficient lactosucrose production and highlights its potential as a cost-effective and food-compatible platform for the synthesis of functional oligosaccharides.

## 1. Introduction

Prebiotics are non-digestible carbohydrates that selectively stimulate the growth and activity of probiotic microorganisms, thereby contributing to host health [1,2] They have been widely incorporated into functional foods and feed formulations, and the global demand continues to rise. However, the consumption of certain prebiotics, such as fructooligosaccharides (FOS) and inulin, is often associated with gastrointestinal discomfort, particularly diarrhea [3]. This limitation has driven the search for alternative prebiotics with improved tolerance. One promising candidate is lactosucrose [O-β-D-galactopyranosyl-(1,4)-O-α-D-glucopyranosyl-(1,2)-β-D-fructofuranoside], a trisaccharide that enhances the growth of some probiotics while producing fewer adverse gastrointestinal effects [4].

Despite its functional benefits, the industrial production of lactosucrose remains challenging. Chemical synthesis is impractical due to low regioselectivity and the formation of unwanted by-products, necessitating extensive downstream purification. Consequently, enzymatic synthesis has become the preferred and most viable production strategy, typically employing lactose as the galactosyl donor and sucrose as the acceptor. To date, only limited number of enzymes including levansucrase, β-fructofuranosidase, and β-galactosidase have been reported to catalyze lactosucrose formation [5]. While levansucrase and β-fructofuranosidase have been extensively investigated, comparatively few studies have focused on lactosucrose synthesis mediated by β-galactosidase, with most reports describing enzymes derived from *Bacillus circulans* [5,6]. However, *B. circulans* is considered an opportunistic pathogen, raising safety concerns for food-related applications. This limitation highlights the need to identify β-galactosidase sources from Generally Recognized As Safe (GRAS) microorganisms, such as *Lactococcus* spp., for consumer-acceptable lactosucrose production [7].

β-Galactosidases from lactic acid bacteria (LAB) are well known for their roles in dairy fermentation and lactose metabolism [8–10]. These enzymes are classified into several glycoside hydrolase (GH) families in the CAZy database [11] The β-galactosidase from *Lactococcus* sp. KTH0-1S characterized in this study belongs to GH family 2, as confirmed by dbCAN2 sequence analysis (E-value = 2 × 10 ^−^ ^268^), consistent with its structural similarity to *Escherichia coli* β-galactosidase (PDB: 1JYX). In addition to lactose hydrolysis, these enzymes are capable of catalyzing transgalactosylation reactions, leading to the formation of lactosucrose as well as galactooligosaccharides (GOS), another class of prebiotics with established health benefits [6,12]. Reaction conditions, particularly high concentrations of lactose and sucrose, favor transgalactosylation over hydrolysis and reduce the formation of undesired by-products [13,14]. Nevertheless, the low native expression levels of β-galactosidases in wild-type LAB significantly restrict their industrial application, as large enzyme quantities are required to achieve economically viable lactosucrose yields. Although recombinant DNA technology enables high-level enzyme production, the use of purified recombinant enzymes in food processing is often accompanied by regulatory and cost-related challenges.

To overcome these limitations, food-grade expression systems based on LAB has been developed [15,16]. Among LAB, *Lactococcus* sp. is particularly attractive due to its GRAS status, long history of safe use in food fermentation, and the availability of well-characterized and high potential molecular tools, including the nisin-controlled gene expression (NICE) system [17–19]. *Lactococcus* sp. KTH0-1S, an indigenous isolate, was used as the expression host. This strain was originally isolated from Thai fermented shrimp (Kung-Som) [19] and was selected as the expression host based on the following rationale: (1) whole-genome sequencing confirmed the absence of antimicrobial resistance genes and virulence factors, supporting its suitability for food-grade applications [20]; (2) the genome encodes the NisRK two-component regulatory system required for nisin-controlled gene expression (NICE), enabling robust inducible protein production; and (3) genomic and phenotypic evidence confirmed a functional respiration metabolism activated by heme supplementation, allowing high-cell-density cultivation and enhanced biomass yield for large-scale biocatalyst preparation [20]. In parallel, advances in whole-cell immobilized technology, especially those employing LysM anchoring domains have enabled the immobilization of heterologous enzymes on the bacterial cell wall [11]. Whole-cell immobilized enzymes offer several advantages over intracellular or free enzyme systems, including elimination of cell disruption and enzyme purification steps, reduced downstream processing costs, enhanced operational stability, reusability, and lower contamination risk [13,21]. LysM (lysin motif) domains are non-catalytic peptidoglycan-binding modules of approximately 40–60 amino acids that mediate non-covalent attachment to N-acetylglucosamine residues in the bacterial cell wall [11].

In this study, we constructed a recombinant *Lactococcus* sp. KTH0-1S strain expressing β-galactosidase using a USP45 signal peptide and a LysM anchoring motif under the control of the NICE system. The engineered strain was evaluated in terms of β-galactosidase activity, induction profile, and its capacity to synthesize lactosucrose from lactose and sucrose at different substrate molar ratios. Furthermore, the stability and catalytic performance of the resulting whole-cell biocatalyst were investigated, along with its potential for reuse in repeated reaction cycles. This work demonstrates the feasibility of employing a GRAS *Lactococcus* host as a safe and efficient whole-cell biocatalysis platform for cost-effective lactosucrose production and provides a foundation for the development of scalable biocatalytic systems for prebiotic synthesis.

## 2. Materials and methods

### 2.1. Bacterial strains and culture conditions

The bacterial strains and plasmids used in this study are summarized in Table 1. *Escherichia coli* DH5α was used as the cloning host and routinely cultivated in Luria–Bertani (LB) medium at 37°C with shaking at 200 rpm. Chloramphenicol was added at a final concentration of 10 µg/mL when required.

**Table 1:** Bacterial strains and plasmids used in this study.

Strain/Plasmid	Relevant characteristics	Reference
*Escherichia coli* DH5α	Cloning host; F– φ80lacZΔM15 Δ(lacZYA-argF)U169 recA1 endA1 hsdR17 (rk–, mk+) phoA supE44 λ– thi-1 gyrA96 relA1	Laboratory stock
*Lactococcus* sp. KTH0-1S	Indigenous isolate; used as expression host for whole-cell β-galactosidase	[19]
pNZ8150	Nisin-inducible expression vector; Cm^R	MoBiTec
pNZ8150-βgal-LysM	pNZ8150 carrying *lacZ* fused with USP45 signal peptide and LysM cell-wall anchor motif for whole cell biocatalysis	This study

*Lactococcus* sp. KTH0-1S, an indigenous isolate, was used as the expression host. Cultures were maintained in M17 broth (Difco) supplemented with 0.5% (w/v) glucose (GM17) and incubated statically at 30°C. For recombinant strains harboring plasmids, chloramphenicol was added at a final concentration of 5 µg/mL. Nisin (MedChemExpress) was used for induction of gene expression as specified in subsequent experiments.

### 2.2. β-Galactosidase activity verification in *Lactococcus* sp. KTH0-1S

#### 2.2.1. Preparation of crude enzyme extracts.

The parental *Lactococcus* sp. KTH0-1S strain was cultivated in GM17 broth at 30°C for 24 h. Starter cultures (50 µL) were inoculated into 5 mL of fresh GM17 medium and incubated under the same conditions. Cells were harvested by centrifugation at 5,000 × g for 15 min at 4 °C, washed twice with phosphate-buffered saline (PBS; 50 mM, pH 6.5), and stored at −20 °C until further use. Cell pellets were resuspended in 2 mL of PBS (50 mM, pH 6.5) and disrupted by sonication [16]. Cell debris was removed by centrifugation at 15,000 × g for 5 min at 4 °C, and the supernatant was collected as the crude enzyme extract.

#### 2.2.2. β-Galactosidase activity assay.

β-Galactosidase activity was measured using o-nitrophenyl-β-D-galactopyranoside (ONPG) as the substrate. The reaction mixture contained 10 µL crude enzyme, 40 µL sodium phosphate buffer (50 mM, pH 6.5), and 250 µL ONPG solution (50 mM). Reactions were incubated at 40 °C and terminated at defined time points (10, 30, 60, and 240 min) by adding 100 µL of 1 M Na_2_CO_3_. Absorbance was measured at 420 nm. Control reactions contained buffer without enzyme. Enzyme activity was expressed as Miller units.

A standard calibration curve was generated using commercial β-galactosidase (Sigma-Aldrich, Germany) at concentrations ranging from 0.1 to 10 U/mL in PBS (50 mM, pH 6.5). Enzyme activity was determined under identical assay conditions with a 30 min incubation at 40 °C. [13].

#### 2.2.3. Lactosucrose synthesis using crude enzyme.

Lactose and sucrose were each dissolved at 500 g/L in 0.1 M sodium phosphate buffer (pH 7.0) and mixed at a 1:1 (v/v) ratio. Reaction mixtures (0.5 mL) were pre-incubated at 30 °C for 30 min, followed by the addition of 0.5 mL crude enzyme extract. Enzymatic reactions were carried out at 50 °C for 4 h and terminated by heating at 100 °C for 5 min. Samples were stored at −20 °C prior to analysis. [13].

### 2.3. Construction of whole-cell recombinant *Lactococcus* sp. expressing β-galactosidase

#### 2.3.1. Plasmid design and construction.

The *lacZ* gene, LysM anchoring domain and USP45 signal peptide were amplified from the *Lactococcus* sp. KTH0-1S genome using specific primers (S1 Table). The expression cassette consisted of an N-terminal USP45 signal peptide for secretion and a C-terminal LysM domain for cell wall anchoring. The assembled fusion into plasmid pNZ8150 using Gibson assembly generating plasmid pNZ8150-USP45-β-gal-LysM. The recombinant plasmid was propagated in *E. coli* DH5α by electroporation and verified by colony PCR, agarose gel electrophoresis, and sequencing. The confirmed construct was subsequently introduced into *Lactococcus* sp. KTH0-1S by electroporation [16,18,22,23].

#### 2.3.2. Induction and whole-cell β-galactosidase activity assay.

Recombinant *Lactococcus* sp. harboring pNZ8150-USP45-β-gal-LysM was cultured in GM17 medium at 30 °C to an OD₆₀₀ of approximately 0.4. Gene expression was induced with nisin at final concentration of 5 ng/mL. Samples were collected at 0, 1, 2, 3, 4, 5 h post-induction and washed twice in Z-buffer (pH 7.0) and resuspended in the same buffer. The whole-cell β-galactosidase activity was measured using ONPG (4 mg/mL in 1 M sodium phosphate buffer, pH 7.0). Reactions were incubated at 28°C for 30 min and terminated by adding 0.5 mL 1 M Na_2_CO_3_. Absorbance was measured at 420 and 550 nm, and enzyme activity was calculated as Miller units.

### 2.4. Expression, extraction, and detection of β-galactosidase

#### 2.4.1. Induction of recombinant protein expression.

*Lactococcus* sp. KTH0-1S wild type (WT) and the recombinant *Lactococcus* sp. KTH0-1S harboring the pNZ8150-USP45-β-gal-LysM construct were cultured in GM17 broth supplemented with 10 µg/mL chloramphenicol (for the recombinant strain) and incubated overnight at 30°C. An aliquot of the overnight culture was sub-cultured (1:25) into fresh GM17 medium and incubated at 30°C until the OD_600_ reached approximately 0.4. Recombinant protein expression was induced by adding nisin to a final concentration of 5 ng/mL, and the induced cultures were incubated at 30°C for 4 h, the optimal induction period based on experiments, with OD_600_ monitored to track cell growth. Cells were then harvested by centrifugation at 6,000 *× g* for 15 min at 4°C and washed twice with 50 mM sodium phosphate buffer (pH 7.0) prior to extraction of cell-associated proteins.

#### 2.4.2. Extraction of cell-associated proteins using LiCl.

Cell-associated proteins were extracted according to the LiCl extraction method. After the final wash, residual buffer was removed and the cell pellet was resuspended in 5 M LiCl at a ratio of 0.1% (w/v) and incubated at 4°C for 1 h with gentle shaking. The suspension was then centrifuged at 22,000 *× g* for 30 min at 4°C, and the resulting supernatant containing the LiCl-extracted proteins was filtered through a 0.2 µm pore-size nitrocellulose membrane. The filtrate was subsequently dialyzed and concentrated using an Amicon® Ultra-15 Centrifugal Filter (10 kDa molecular weight cut-off; Millipore, USA) by centrifugation at 5,000 *× g* for 30 min at 4°C. Protein concentration in the LiCl extracts was determined using Bradford assay.

#### 2.4.3. Dot blot analysis.

To compare protein expression between *Lactococcus* sp. KTH0-1S WT and the recombinant strain, LiCl-extracted protein samples were detected using dot blot method. A nitrocellulose membrane was mounted onto a dot blot apparatus (Bio-Dot® Microfiltration Apparatus, Bio-Rad Laboratories), and 5 µg protein of each sample was spotted onto the membrane, which had been pre-treated with PBST (0.1% (v/v) Tween 20 in 1 × PBS). The membrane was air-dried at room temperature for at least 60 min to ensure complete protein adsorption before blocking. The membrane was then blocked with 5% (w/v) skim milk in PBST for 60 min at room temperature and washed three times with PBST for 10 min each. The membrane was incubated with anti-His primary antibody (1:1000 dilution in blocking solution) for 2 nights at 4°C, followed by three washes with PBST (10 min each). The membrane was subsequently incubated with anti-IgG-HRP secondary antibody (1:1000 dilution in blocking solution) for 2 h at room temperature with shaking, then washed once with PBST followed by three washes with PBS (10 min each). Bound antibody was visualized by overlaying the membrane with developing solution (0.1 M NaCl, 5 mM MgCl2, 0.1 M Tris-HCl, pH 9.5) for 10 min, followed by incubation with 2 mL of ECL substrate in the dark for approximately 5 min, and signals were detected using a GelDoc XR imaging system (Bio-Rad Laboratories, Hercules, CA, USA).

### 2.5. Effect of pH and temperature on lactosucrose synthesis

The effect of pH on lactosucrose synthesis was examined using buffers at different pH values: acetate buffer (50 mM, pH 5.0–6.0), phosphate buffer (50 mM, pH 6.0–8.0), and Tris–HCl buffer (50 mM, pH 8.0–9.0) [21]. Lactose and sucrose were each added at 12.5% (w/v). Reactions were performed with recombinant *Lactococcus* sp. cells at a final OD₆₀₀ of 20 and incubated at 50 °C for 4 or 24 h.

For temperature optimization, reactions were conducted in acetate buffer (50 mM, pH 6.0) at 30, 40, 50, and 60 °C for 4, 8, 24, and 48 h using recombinant cells at a final OD₆₀₀ of 100.

### 2.6. Effect of substrate molar ratio on lactosucrose production and conversion cycle of the whole-cell immobilized *Lactococcus* sp.

To evaluate the effect of substrate molar ratio, lactose and sucrose were prepared in acetate buffer (50 mM, pH 6.0) at molar ratios of 3:1, 2:1, 1:1, 1:2, and 1:3 (lactose:sucrose). Reactions were carried out under optimized conditions at 30, 40, and 50 °C for 24 h using whole-cell biocatalyst recombinant *Lactococcus* sp. with β-galactosidase (final OD₆₀₀ = 100). Reactions were terminated by heating at 100 °C for 5 min.

Reusability of the whole-cell biocatalyst was assessed over two consecutive reaction cycles. After each 24 h reaction, cells were recovered by centrifugation, washed with buffer, and reused under identical conditions. Lactosucrose production was quantified after each cycle by HPLC.

### 2.7. Sugar Analysis by HPLC

Reaction samples were filtered through 0.22 µm membranes prior to HPLC analysis. Sugars were separated using an Aminex HPX-87C column (Bio-Rad; 300 × 7.8 mm) equipped with a refractive index detector (Agilent, USA). Deionized water was used as the mobile phase at a flow rate of 0.2 mL/min, and the column temperature was maintained at 85 °C. Lactosucrose, lactose, sucrose, glucose, galactose, and fructose were quantified by comparison with authentic standards.

### 2.8. Statistical analysis

All experiments were performed in **biological triplicate**, and each measurement was conducted in **technical triplicate** unless otherwise stated. Results are presented as the **mean** ± standard deviation (SD). Statistical analyses were performed using SPSS **software (version 22)**.

Comparisons between two groups were conducted using **Student’s** *t***-test**, while comparisons among multiple groups were analyzed using **one-way analysis of variance (**ANOVA) followed by **Tukey’s post hoc test**. Differences were considered statistically significant at *p* < 0.05.

For optimization studies involving pH, temperature, reaction time, substrate molar ratio, and reusability cycles, statistical significance was assessed by ANOVA across experimental conditions. All statistical tests were **two-tailed**.

### 2.9. *In silico* Structural Modelling and Docking Analysis of β-Galactosidase from *Lactococcus* sp. KTH0-1S

The oligomeric structure of β-galactosidase from *Lactococcus* sp. KTH0-1S was predicted using AlphaFold3 [24]. To identify the key residues within the active site, the predicted structure of *Lactococcus* sp. KTH0-1S β-galactosidase was aligned with the crystal structure of *E. coli* β-galactosidase contain IPTG (PDB: 1JYX) using the PyMOL Molecular Graphics System (version 2.4, Schrödinger, LLC). After alignment, IPTG was extracted as an object. The structure of *Lactococcus* sp. KTH0-1S β-galactosidase was visualized with only IPTG shown, while the protein template was hidden to facilitate better understanding.

In addition, a structural model of the enzyme was generated using the SWISS-MODEL protein modeling server (https://swissmodel.expasy.org/). The chemical structures of lactose, galactose, and sucrose were retrieved from the PubChem database (https://pubchem.ncbi.nlm.nih.gov/), and all relevant cofactors were included in docking simulations. Automated molecular docking was carried out using the CB-Dock2 web server (https://cadd.labshare.cn/cb-dock2/index.php). Docking results were analyzed to identify amino acid residues involved in ligand binding, including residues common to all ligands and those specific to individual interactions. The protein sequence of β-galactosidase from *Lactococcus* sp. KTH0-1S was subjected to BLAST analysis (blastp) against the NCBI non-redundant protein database restricted to the genus Lactococcus to assess sequence conservation across related species. GH family classification was performed using the dbCAN2 server (https://bcb.unl.edu/dbCAN2/) with hmmscan against the CAZy database

## 3. Result

### 3.1. Determination of intracellular β-Galactosidase activity in wild-type *Lactococcus* sp. KTH0-1S

Intracellular β-galactosidase activity of wild-type *Lactococcus* sp. KTH0-1S was determined in crude cell extracts using o-nitrophenyl-β-D-galactopyranoside (ONPG) as the substrate. The absorbance at 420 nm increased progressively with incubation time, with OD_420_ values of 0.171, 1.533, 2.294, and 3.139 recorded at 10, 30, 60, and 240 min, respectively (S1A Fig). The reaction at 10 min was insufficient for accurate quantification, whereas reactions at 60 and 240 min deviated from linearity, likely due to substrate depletion or product inhibition. Based on the linear reaction phase, a 30 min incubation was selected as the optimal assay condition for subsequent measurements. Enzyme activity under this condition was calculated as 10.15 U/mL using a standard curve generated with commercial β-galactosidase (S1B Fig).

### 3.2. Lactosucrose synthesis capability of *Lactococcus* sp. KTH0-1S

HPLC analysis confirmed that crude β-galactosidase extracted from wild-type *Lactococcus* sp. KTH0-1S efficiently catalyzed lactosucrose synthesis from lactose and sucrose at 50 °C. After 4 hours of incubation, lactosucrose concentration reached 3,740 ± 310 mg/L and increased significantly to 6,280 ± 220 mg/L when the reaction time was extended to 4.5 h (*p* < 0.05). No lactosucrose formation was detected in enzyme-free control. (Table 2).

**Table 2:** Lactosucrose production by crude β-galactosidase from wild-type *Lactococcus* sp. KTH0-1S.

Sample	Reaction time (h)	Lactosucrose (mg/L)
Conversion 1	4	3,740 ± 310
Conversion 2	4.5	6,280 ± 220
Control	4.5	0

Note: Values represent the mean ± SD of three independent experiments (*n* = 3). “Conversion 1” and “Conversion 2” represent independent reactions conducted at different incubation times (4 h and 4.5 h, respectively), not technical replicates of the same condition.

This result demonstrated that β-galactosidase from *L. lactis* KTH0-1S possesses intrinsic transgalactosylation activity and is capable of producing lactosucrose under the tested conditions. Based on this validated catalytic capability, the corresponding β-galactosidase gene was selected for subsequent cloning and development of a whole-cell recombinant system.

Moreover, the enzyme exhibited thermal stability at 50°C for extended periods, maintaining catalytic activity throughout the reaction period. The time-dependent increase in lactosucrose yield indicated a direct correlation between incubation time and product formation. These results suggest that enzyme activity and substrate availability both influence synthesis rates, with non-uniform production rates observed across different time intervals. The findings demonstrate that reaction time and substrate concentration are critical parameters affecting lactosucrose synthesis efficiency. Further optimization studies using immobilized cell systems are warranted to determine optimal reaction conditions.

### 3.3. Design of the β-galactosidase expression construct for whole-cell immobilized Lactococcus sp.

A recombinant expression construct was designed to enable whole-cell biocatalysis of β-galactosidase in *Lactococcus* sp. KTH0-1S. The construct consisted of an N-terminal USP45 signal peptide (358 bp) to direct protein secretion, followed by the *lacZ* gene (2,996 bp) encoding β-galactosidase, and a C-terminal LysM (lysin motif) cell wall anchoring domain to facilitate surface attachment. The *lacZ* gene was identified at genomic coordinates 2,064,341–2,067,337 based on KEGG database analysis. The encoded β-galactosidase was classified into glycoside hydrolase (GH) family 2 based on sequence analysis using the dbCAN2 server (E-value = 2 × 10 ^−^ ^268^, coverage = 98.8%). BLAST analysis against the NCBI non-redundant protein database restricted to the genus *Lactococcus* revealed highest sequence identity to β-galactosidase from *Lactococcus lactis* (97.19%, 100% query coverage; WP_404378437.1), followed by *Lactococcus kimchii* (69.32%), *Lactococcus taiwanensis* (64.04%), *Lactococcus hircilactis* (58.79%), and *Lactococcus termiticola* (55.69%). Gibson assembly primers containing overlapping regions were designed to assemble the USP45–*lacZ*–LysM fusion cassette into the expression vector by homologous recombination (S1 Table). Successful insertion of the expression cassette into pNZ8150 was confirmed by colony PCR using construct-specific primers, which yielded a band of the expected size (~3,648 bp) in all screened colonies (S2 Fig). The correct sequence of the cloned cassette was further verified by Sanger sequencing, and the full nucleotide sequence of the USP45–lacZ–LysM expression cassette is provided in S1 File.

### 3.4. Prediction of protein localization

Subcellular localization of the β-galactosidase constructs was predicted using DeepLocPro 1.0 (Table 3). The native β-galactosidase from *Lactococcus* sp. KTH0-1S was predicted to be predominantly cytoplasmic, with a localization probability of 0.955, consistent with the observed intracellular enzyme activity. Incorporation of the USP45 signal peptide alone markedly increased the predicted extracellular localization probability to 0.669, while reducing cytoplasmic localization to 0.243. Addition of the LysM anchoring domain further increased the probability of cell wall association (0.147) while maintaining substantial extracellular localization (0.467). Notably, the complete USP45–β-galactosidase–LysM construct exhibited the most favorable surface display profile, characterized by a high extracellular localization probability (0.832), enhanced cell wall association (0.152), and minimal cytoplasmic retention (0.014). **These** *in silico* **predictions were subsequently validated by experimental measurements of whole-cell** β-**galactosidase activity.**

**Table 3:** Predicted subcellular localization probabilities of β-galactosidase constructs in *Lactococcus* sp. KTH0-1S using DeepLocPro 1.0. Values represent the predicted likelihood of protein localization to each cellular compartment.

Sequence	Localization					
	Cell wall & surface	Extracellular	Cytoplasmic	Cytoplasmic Membrane	Outer Membrane	Periplasmic
Native β-galactosidase	0.0019	0.0408	0.9550	0.0023	0.0000	0.0000
USP45- β-gal	0.0786	0.6689	0.2431	0.0093	0.0000	0.0000
β-gal-CWA	0.1471	0.4665	0.3796	0.0068	0.0000	0.0000
USP45-β-gal-CWA	0.1516	0.8317	0.0138	0.0029	0.0000	0.0000

### 3.5. Screening of recombinant *Lactococcus* sp. clones for whole-cell immobilized β-galactosidase activity

**Fig 1:**
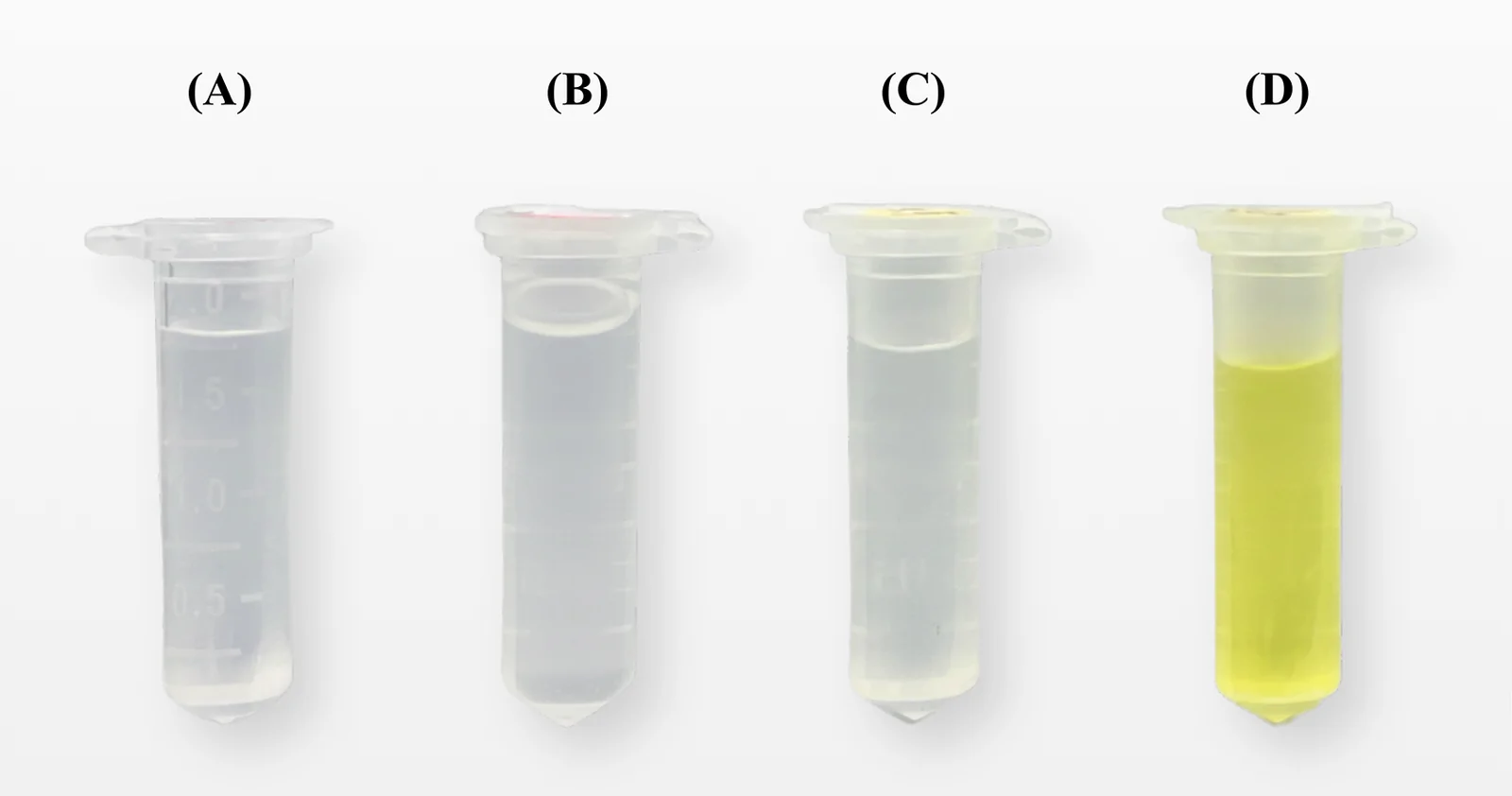
β-galactosidase activity measurement using ONPG (A) negative control, (B) Lactococcus sp. KTH0-1S (wild-type), (C) Lactococcus sp. Clone 2 (non-induced), (D) Lactococcus sp. Clone 2 (induced).

Recombinant *Lactococcus* sp. KTH0-1S clones harboring the expression construct (USP45–β-galactosidase–LysM) were successfully obtained and screened for β-galactosidase activity on the whole-cell. Two representative clones were selected for further characterization. Enzymatic activity was evaluated under both induced and non-induced conditions using nisin as the inducer. No detectable β-galactosidase activity was observed using whole-cell of the wild-type *Lactococcus* sp. KTH0-1S (Fig 1B) or in non-induced recombinant strains (Fig 1C), indicating the absence of leaky expression.

Upon nisin induction, both recombinant clones exhibited a marked increase in whole-cell β-galactosidase activity compared with the corresponding non-induced controls (Fig 1D; Table 4). Among the tested clones, Clone 2 showed the highest enzymatic activity, reaching 146.0 ± 4.5 Miller Units, which was significantly higher than that of Clone 1 (*p* < 0.05). In addition, Clone 2 reached maximal activity more rapidly (25 min) than Clone 1 (50 min), indicating enhanced catalytic performance. Based on its superior activity and faster reaction kinetics, Clone 2 was selected for subsequent studies on recombinant β-galactosidase expression and lactosucrose synthesis.

**Table 4:** Whole-cell β-galactosidase activity of recombinant *Lactococcus* sp. KTH0-1S clones under induced and non-induced conditions.

Clone	Condition	β-Galactosidase activity (Miller Units)	Time to reach maximum activity (min)
Clone 1	Non-induced	ND (not detected)	–
Clone 1	Induced	98.4 ± 3.2^a^	50
Clone 2	Non-induced	ND (not detected)	–
Clone 2	Induced	**146.0** ± **4.5**^**b**^	25

Note: Values represent the mean ± SD of three independent experiments (*n* = 3). ND = not detected.

### 3.6. Effect of induction time on the activity of whole-cell β-galactosidase in recombinant *Lactococcus* sp

**Fig 2:**
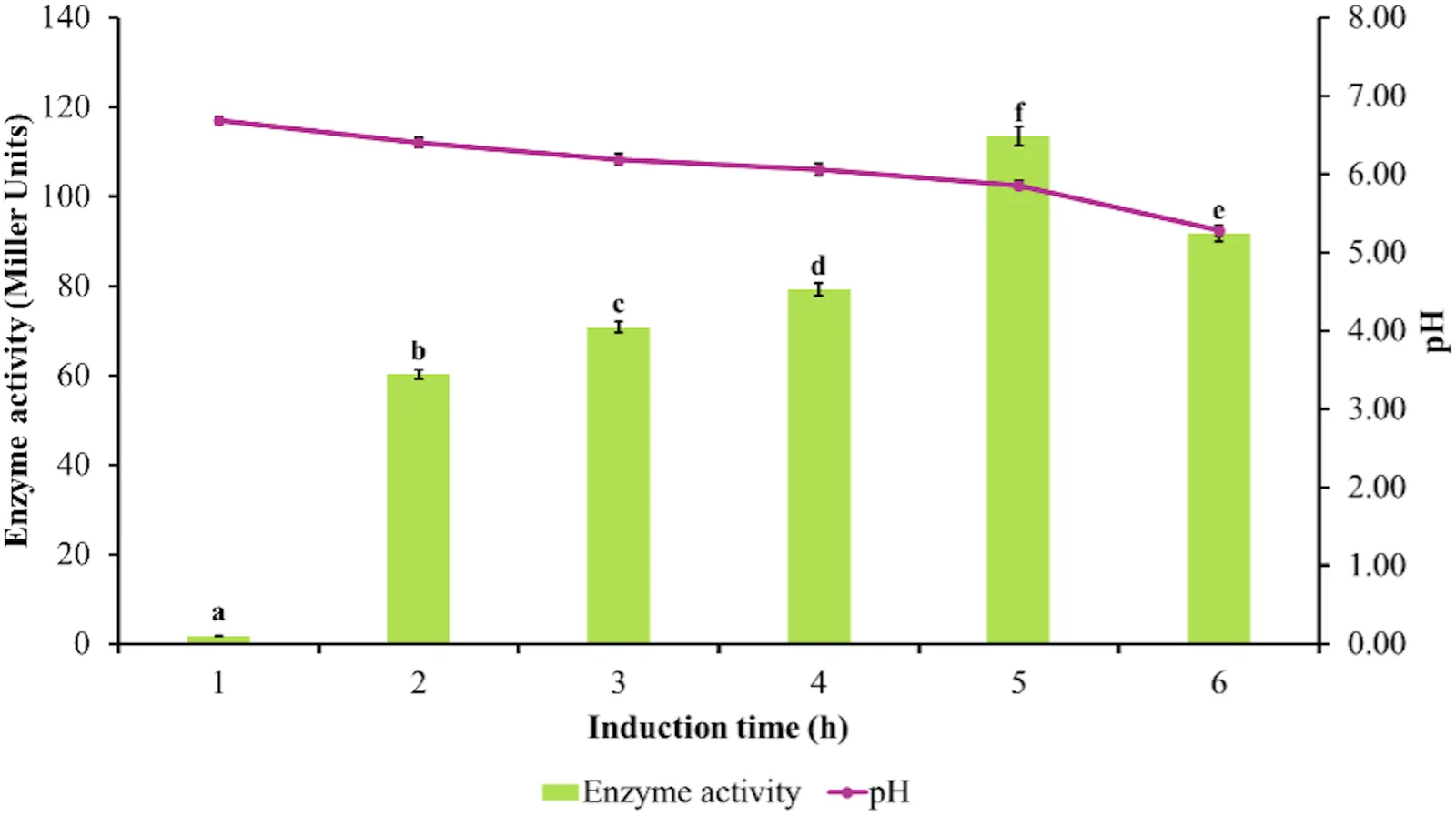
Effect of induction time on whole-cell β-galactosidase activity and culture pH in recombinant Lactococcus sp. KTH0-1S. Cells harboring the USP45–β-galactosidase–LysM construct were induced with nisin and sampled at the indicated time points. β-Galactosidase activity (bars) is expressed as Miller Units, while culture pH (line) was monitored concurrently during induction. Data represent the mean ± SD of three independent experiments (*n* = 3). A significant decrease in enzyme activity was observed after 5 h of induction (*p* < 0.05).

The effect of induction time on the activity of whole-cell β-galactosidase in recombinant *Lactococcus* sp. KTH0-1S was evaluated under constant cell density and assay conditions to allow direct comparison of enzyme activity. Following nisin induction, the culture pH gradually decreased from 6.69 to 5.86 during the first 4 h and further declined to 5.28 after 5 h of induction (Fig 2). Whole-cell β-galactosidase activity increased progressively during the early induction period (1–4 h), reaching a maximum value of 113.45 ± SD Miller Units at 4 h (Fig 2). Prolonged induction to 5 h resulted in a significant reduction in enzyme activity (*p* < 0.05), indicating that extended induction adversely affected surface enzyme performance. Based on these results, an induction time of 4 h was selected as optimal for subsequent experiments.

### 3.7. Effect of pH on the activity of whole-cell β-galactosidase

**Fig 3:**
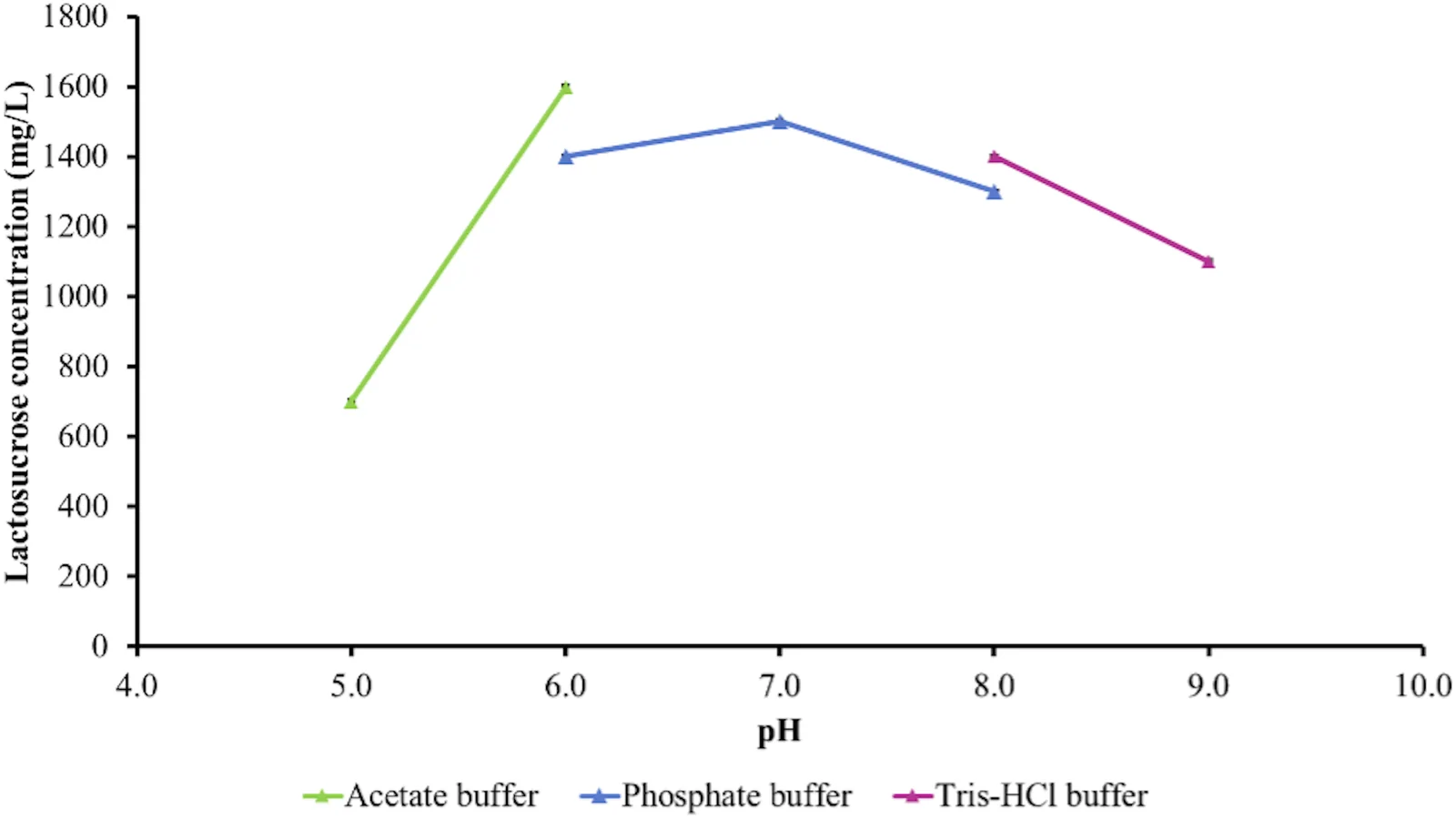
Effect of reaction pH on lactosucrose synthesis catalyzed by whole-cell β-galactosidase in recombinant Lactococcus sp. KTH0-1S. Lactosucrose production was evaluated in acetate buffer (pH 5.0–6.0), phosphate buffer (pH 6.0–8.0), and Tris–HCl buffer (pH 8.0–9.0) under otherwise identical reaction conditions (recombinant *Lactococcus* sp. KTH0-1S cells at OD_600_ = 20; lactose and sucrose each at 12.5% w/v; 50 °C; 4 h incubation). Lactosucrose concentration was quantified by HPLC. Data represent the mean ± SD of three independent experiments (*n* = 3).

The effect of pH on the catalytic activity of whole-cell β-galactosidase from recombinant *Lactococcus* sp. KTH0-1S was evaluated during lactosucrose synthesis. Reaction pH had a significant influence on enzyme activity. The highest lactosucrose production (1,600 mg/L) was observed in acetate buffer at pH 6.0 (Fig 3). Comparable lactosucrose yields (1,300–1,600 mg/L) were obtained in phosphate buffer across pH 6.0–8.0 and in Tris–HCl buffer at pH 8.0, with no statistically significant differences among these conditions (*p* > 0.05).

In contrast, enzyme performance declined markedly at more extreme pH values. Lactosucrose production decreased to 1,100 mg/L in Tris–HCl buffer at pH 9.0, likely due to reduced enzyme stability under alkaline conditions. Similarly, acetate buffer at pH 5.0 resulted in the lowest lactosucrose yield (700 mg/L). This reduction may be attributed to the predicted isoelectric point (pI = 5.0) of β-galactosidase, at which protein aggregation or precipitation can occur, leading to reduced catalytic efficiency. Collectively, these results indicate that the optimal pH range for lactosucrose synthesis using whole-cell β-galactosidase in *Lactococcus* sp. lies between pH 6.0 and 8.0.

### 3.8. Effect of temperature and reaction time on β-galactosidase activity

**Fig 4:**
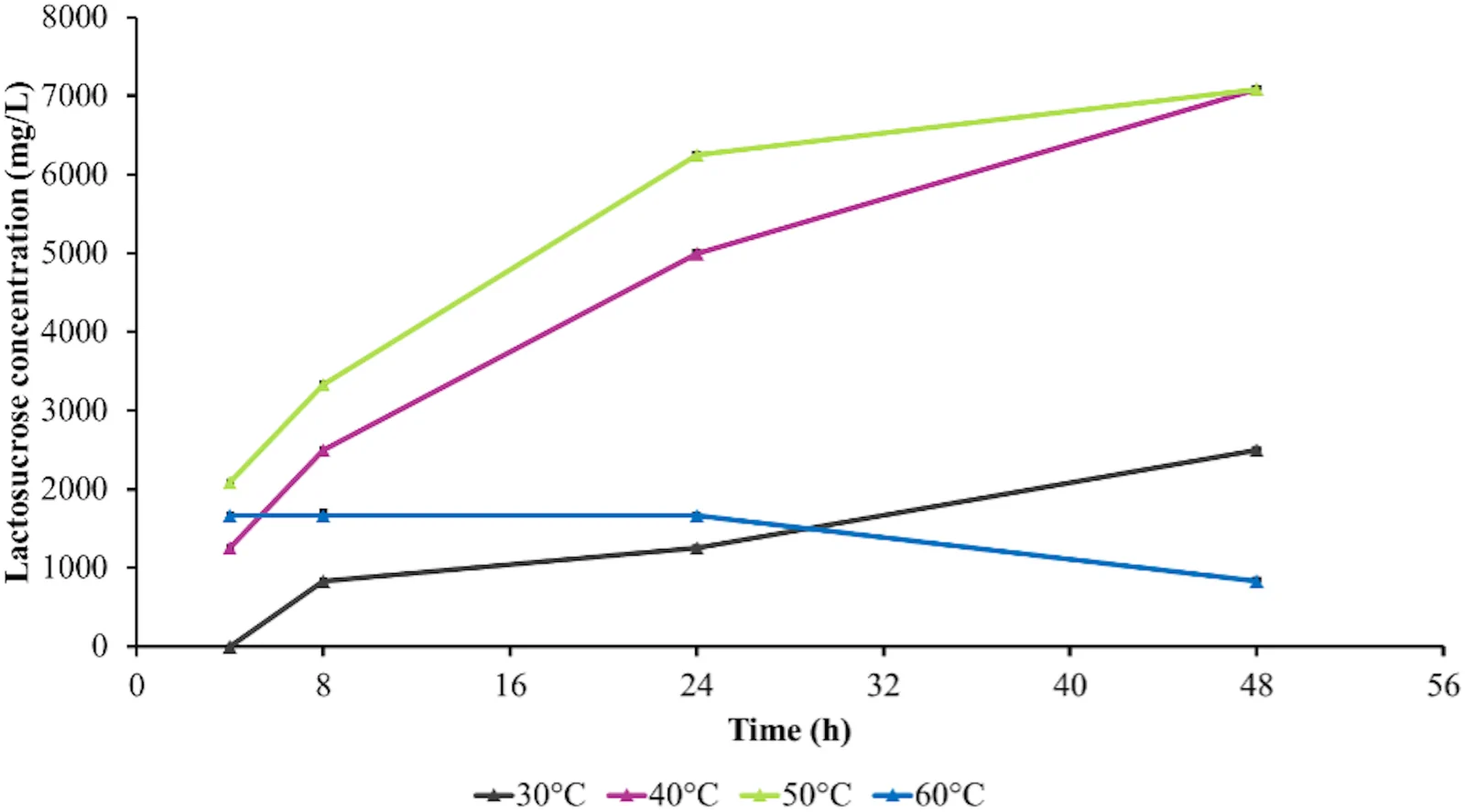
Effect of temperature and reaction time on lactosucrose production catalyzed by whole-cell β-galactosidase in recombinant Lactococcus sp. KTH0-1S. Lactosucrose synthesis was evaluated at 30, 40, 50, and 60 °C over reaction times ranging from 4 to 48 h using lactose and sucrose as substrates. Lactosucrose concentration was quantified by HPLC. Data represent the mean ± SD of three independent experiments (*n* = 3).

The effect of temperature on lactosucrose synthesis catalyzed by whole-cell β-galactosidase in recombinant *Lactococcus* sp. KTH0-1S was evaluated at 30, 40, 50, and 60 °C. At a fixed reaction time of 4 h, lactosucrose production increased with temperature up to 50 °C, yielding 0, 1, 300, and 2,100 mg/L at 30, 40, and 50 °C, respectively (Fig 4). In contrast, lactosucrose production decreased to 1,700 mg/L at 60 °C, indicating reduced catalytic performance at elevated temperature.

The influence of reaction time was subsequently examined at different temperatures. At 30–50 °C, lactosucrose synthesis increased progressively up to 24 h and then reached a plateau between 24 and 48 h, suggesting limitations imposed by substrate availability or product accumulation at extended reaction times. At 60 °C, lactosucrose levels remained relatively constant (approximately 1,700 mg/L) between 4 and 24 h but declined markedly to 800 mg/L after 48 h, indicating substantial loss of enzymatic activity under prolonged high-temperature conditions. Collectively, these results identify 50 °C as the optimal temperature for lactosucrose synthesis using whole-cell β-galactosidase under the tested conditions.

### 3.9. Effect of substrate molar ratio and reusability on lactosucrose production

The molar ratio of lactose to sucrose significantly affected lactosucrose synthesis catalyzed by whole-cell β-galactosidase in recombinant *Lactococcus* sp. KTH0-1S (Table 5). At an equimolar ratio (1:1), the lowest lactosucrose concentration was obtained (4,110 ± 105 mg/L). Increasing the proportion of either substrate enhanced lactosucrose production, yielding 6,520 ± 475 mg/L at a 2:1 ratio and 7,770 ± 197 mg/L at a 1:2 ratio. Further increases in substrate imbalance led to substantially higher production, with lactosucrose concentrations of 14,680 ± 150 mg/L at a 3:1 ratio and a maximum of 15,940 ± 513 mg/L at a 1:3 ratio (*p* < 0.05). These results indicate that sucrose enrichment provides the most favorable condition for lactosucrose formation under the tested conditions. The identity of lactosucrose produced under the optimal substrate ratio (lactose:sucrose = 1:3) was confirmed by HPLC analysis, where the product peak co-eluted with an authentic lactosucrose standard at a retention time of ~22.1 min (S3 Fig). The chromatogram further revealed a corresponding decrease in residual sucrose and lactose peaks relative to the standard mixture, consistent with substrate consumption during enzymatic transglycosylation.

**Table 5:** Effect of lactose:sucrose molar ratio on lactosucrose production and reusability of whole-cell β-galactosidase in recombinant *Lactococcus* sp. KTH0-1S.

Molar ratio Lactose:Sucrose	Conversion cycle	Lactosucrose conc. (mg/L)
	1	14,680 ± 150.00^h^
3:1	2	1,370 ± 25.00^c^
	3	–
	1	6,520 ± 475.00^f^
2:1	2	1,110 ± 100.00^b^
	3	–
	1	4,110 ± 105.00^e^
1:1	2	720 ± 20.00^a^
	3	–
	1	7,770 ± 197.00^g^
1:2	2	1,060 ± 0.00^b^
	3	–
	1	15,940 ± 513.00^i^
1:3	2	2,030 ± 152.00^d^
	3	–

Values represent the mean ± SD of three independent experiments (*n* = 3). Different superscript letters within the same column indicate statistically significant differences (*p* < 0.05). “–” indicates no detectable lactosucrose.

The reusability of the whole-cell biocatalyst was subsequently evaluated over consecutive conversion cycles. In the first cycle, high lactosucrose yields were observed across all substrate ratios (Table 5). In the second cycle, lactosucrose production declined markedly to approximately 700–2,000 mg/L, corresponding to 10–20% of the initial yield. The extent of activity loss varied with substrate ratio, with the smallest reduction observed at a 1:1 ratio, whereas more pronounced decreases were detected at higher imbalance ratios (3:1 and 1:3). No lactosucrose production was detected in the third conversion cycle under any condition, indicating complete loss of catalytic activity.

### 3.10. Structural and Preliminary Characterization of β-galactosidase from *Lactococcus* sp. KTH0-1S

**Fig 5:**
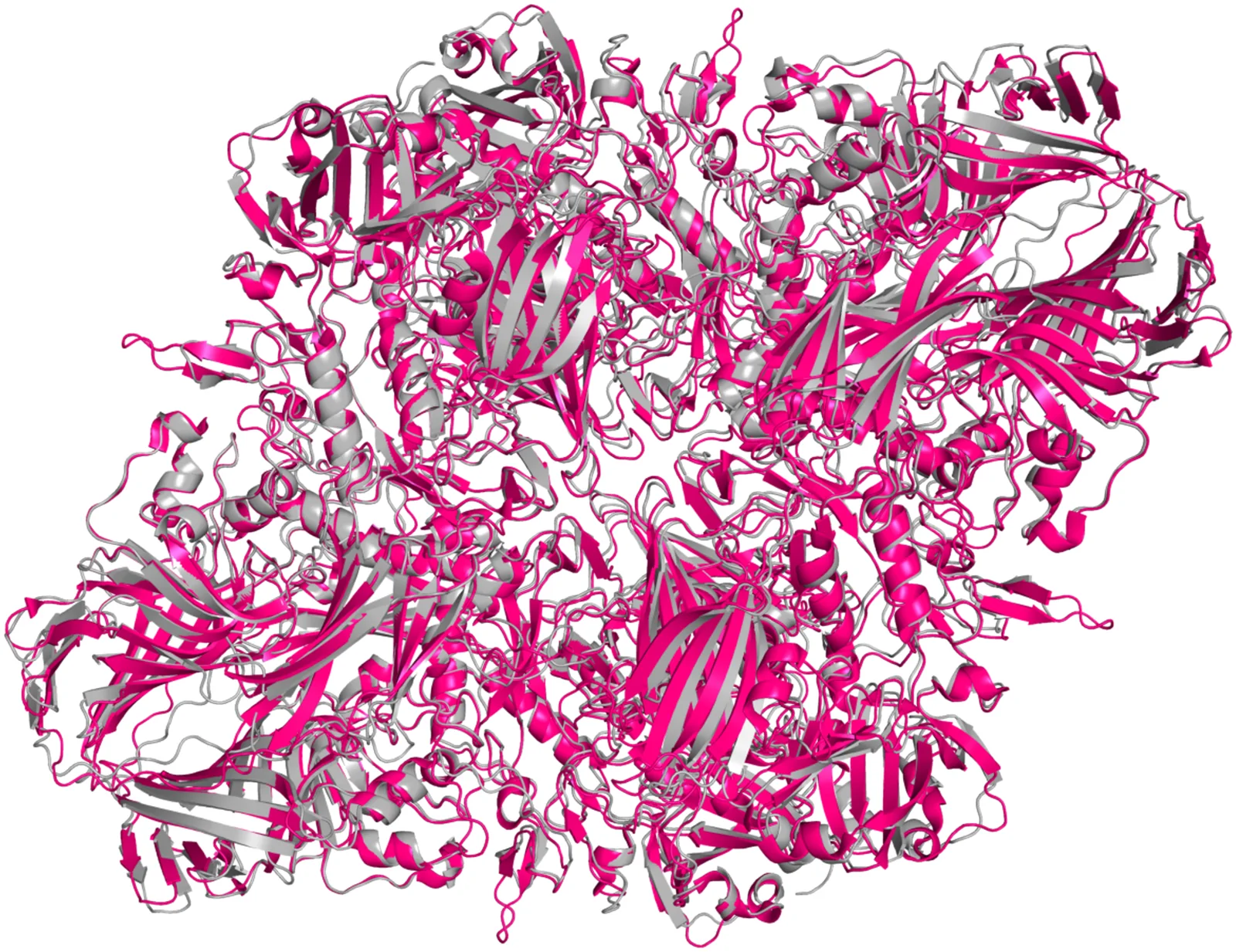
Structural comparison of predicted β-galactosidase from *Lactococcus* sp. KTH0-1S (gray structure) with β-galactosidase from *E. coli* 1JYX (pink structure) reported in the database.

**Fig 6:**
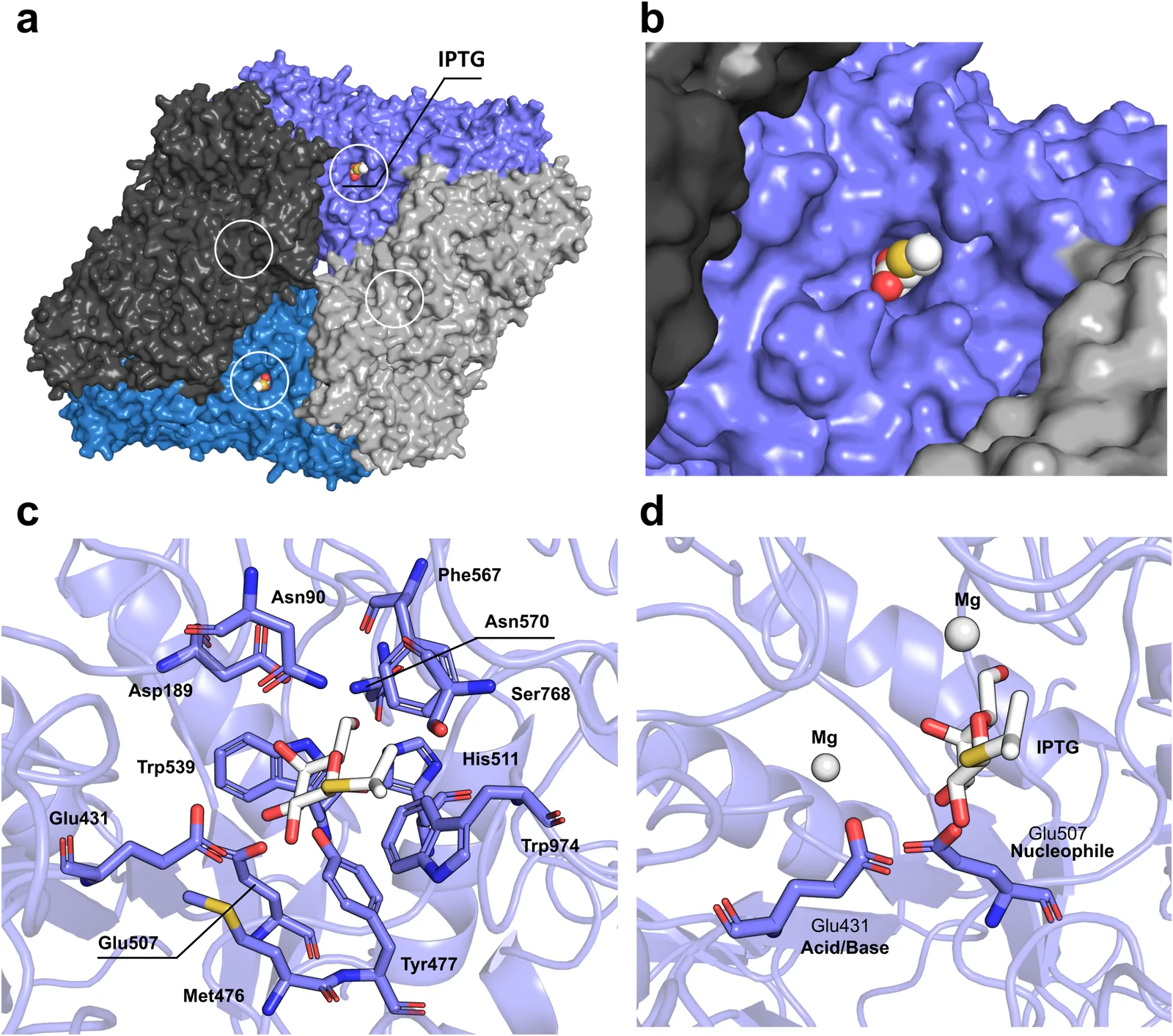
Protein structure of β-galactosidase from *Lactococcus* sp. KTH0-1S; A: AlphaFold-predicted tetrameric structure of β-galactosidase overlaid with the *E. coli* LacZ template containing IPTG as a ligand; different colors represent individual subunits. The active site is present in each subunit showing in the white circle; B: Surface-exposed active site of β-galactosidase from *Lactococcus* sp. KTH0-1S; C: Superposition of active-site residues located within 5 Å of the ligand, highlighting residues involved in sugar binding and catalysis; D: Catalytic residues include Glu431 and Glu507, proposed to function as the acid/base and nucleophile residues, respectively, along with a magnesium ion.

Based on protein structure modeling by AlphaFold3, β-galactosidase from *Lactococcus* sp. KTH0-1S is predicted to be a homotetramer, as shown in Fig 5, which corresponds to the crystal structure of *E. coli* β-galactosidase (β-galactosidase; PDB: 1JYX) containing IPTG molecules [25]. *E. coli* β-galactosidase was selected as the template because it provides 98% query coverage and 50% identity with β-galactosidase from *Lactococcus* sp. KTH0-1S (S2 Table). A structural overlay of these two proteins was performed using the PyMOL Molecular Graphics System (Schrödinger, LLC), yielding an RMSD value of 0.462, indicating a nearly identical overall structure between *Lactococcus* sp. KTH0-1S β-galactosidase and *E. coli* β-galactosidase. The IPTG molecules, which serve as ligands in the template protein, were located at the surface of *Lactococcus* sp. KTH0-1S β-galactosidase, suggesting a surface-exposed active site (Fig 6A, B).

To identify the crucial residues in the active site of *Lactococcus* sp. KTH0-1S β-galactosidase, residues located within 5 Å of the IPTG molecule were examined (Fig 6C). Among these amino acids expected to be involved in sugar binding, Glu431 and Glu507 are proposed to act as the acid/base and nucleophile residues, respectively (Fig 6D). Moreover, a magnesium ion was also observed in the active site, suggesting that it plays an important role in catalysis.

To further investigate substrate recognition, molecular docking of lactose, sucrose, and galactose to β-galactosidase from *Lactococcus* sp. KTH0-1S was performed. Docking analyses focused on Pocket 3 of Chain A, which consistently yielded the most favorable binding poses among the predicted cavities. To provide quantitative support for the docking analysis, the best AutoDock Vina binding energies for each ligand in Pocket 3 were calculated and are reported**,** allowing comparison of relative binding favorability among substrates. The best AutoDock Vina binding scores for Pocket 3 were −**6.8 kcal/mol for lactose,** −**7.1 kcal/mol for sucrose,** and −7.1 kcal/mol for galactose (Table 6).

**Table 6:** Amino acid residues in Pocket 3 of Chain A of β-galactosidase from *Lactococcus* sp. KTH0-1S predicted to interact with lactose, sucrose, and galactose based on molecular docking analysis.

Ligand (substrate)	Best Vina score (kcal/mol)	Interacting residues
**Lactose**	−**6.8**	ASN90, VAL91, HIS388, GLU431, MET476, TYR477, TRP974, ASP774, SER975
**Sucrose**	−**7.1**	ASN90, VAL91, ASP189, HIS388, GLU431, MET476, TYR477, TRP974, GLU508, HIS511
**Galactose**	−**7.1**	ASN90, HIS361, ASP189, GLU386, HIS388, ASN430, GLU431, MET476, TYR477, TRP974, GLU508, HIS511, TRP539, PHE567

**Fig 7:**
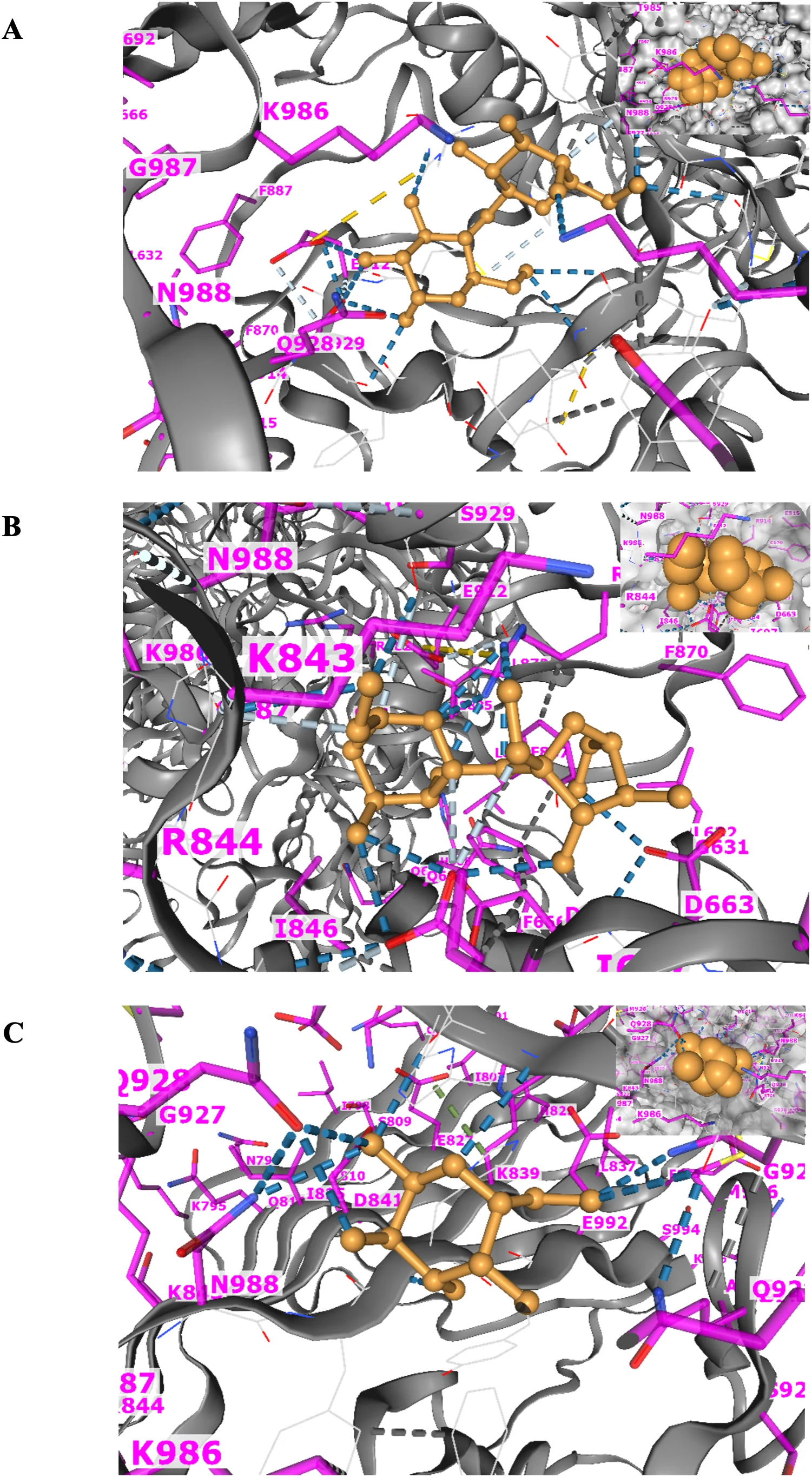
Molecular docking of sugar substrates (A: lactose, B: sucrose, C: galactose) into the β-galactosidase structure of *Lactococcus* sp. KTH0-1S**.** The specific molecular contacts are color-coded as follows, gold ring represent substrate, dotted yellow line represent ionic bond, dotted blue line represent hydrogen bond, dotted white line represent weak hydrogen bond and dotted gray line represent hydrophobic contact**.**

Several amino acid residues—ASN90, HIS388, GLU431, MET476, TYR477, and TRP974 were common to all ligand–enzyme complexes, indicating the presence of a conserved binding core within Pocket 3. Additional overlapping interactions were observed between lactose and sucrose at VAL91 (Fig 7 A, B), as well as between galactose and sucrose at ASP189, GLU508, and HIS511 (Fig 7 B, C), suggesting partially shared binding features that may facilitate both hydrolytic and transgalactosylation reactions.

### 3.11 Confirmation of whole-cell β-galactosidase expression by dot blot

**Fig 8:**
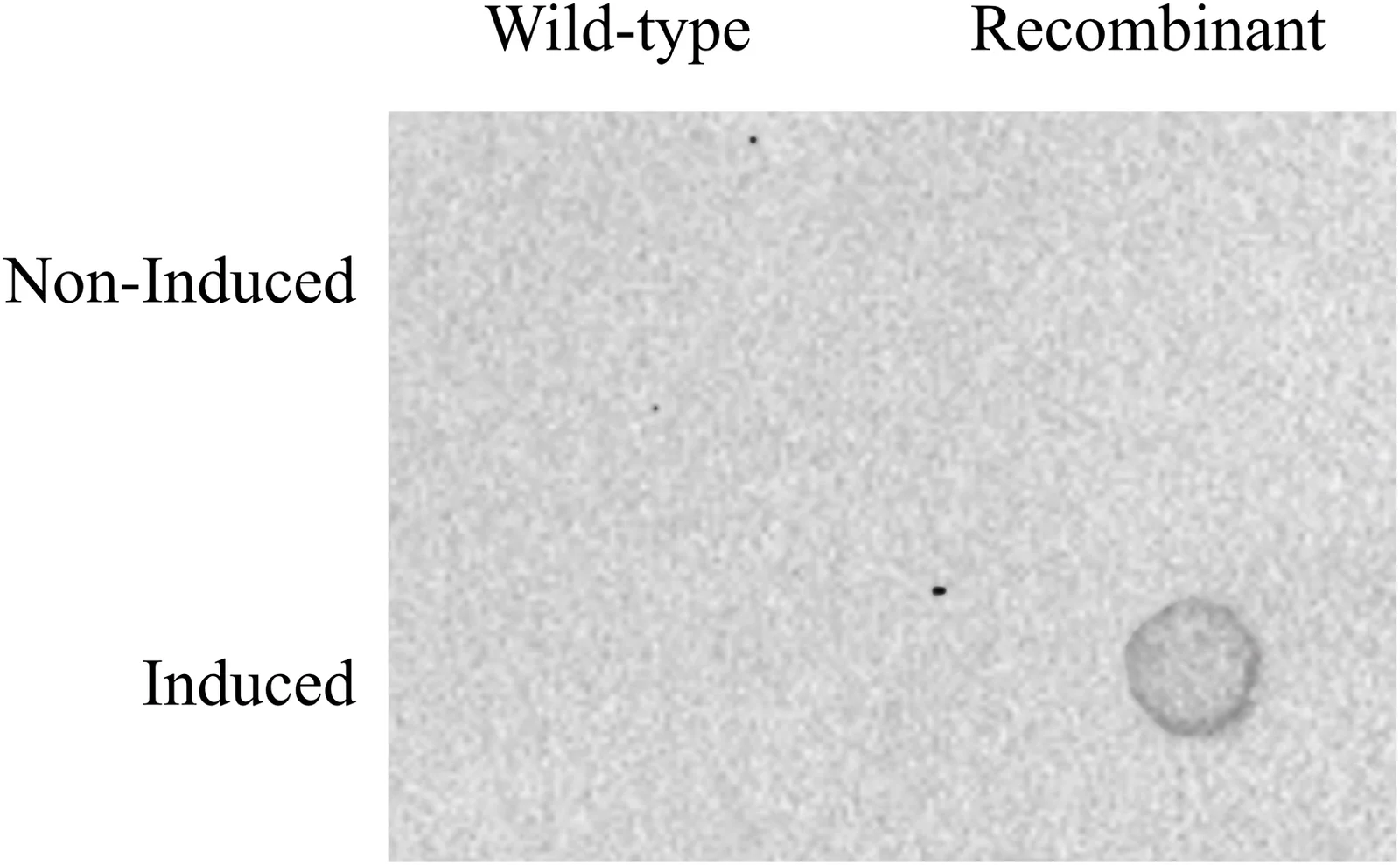
Dot blot analysis of whole-cell β-galactosidase in Lactococcus sp. KTH**0-1S.** LiCl-extracted cell associated protein fractions from wild-type (WT) and recombinant *Lactococcus* sp. KTH0-1S, under non-induced and nisin-induced conditions.

To confirm whole-cell expression of the recombinant β-galactosidase, LiCl-extracted protein fractions from *Lactococcus* sp. KTH0-1S WT and recombinant strains, both non-induced and nisin-induced (4 h), were analyzed by dot blot using an anti-His antibody (Fig. 8). A distinct immunoreactive signal was detected only in the LiCl extract from the induced recombinant strain, whereas no signal was observed in the non-induced recombinant sample or in either WT sample (non-induced and induced). These results confirm that the His-tagged β-galactosidase was successfully expressed of the recombinant *Lactococcus* sp. KTH0-1S, and that its expression was dependent on nisin induction, consistent with the β-galactosidase activity on whole cell analysis. The absence of signal in the WT strain further confirms the specificity of the anti-His antibody and rules out cross-reactivity with native cell.

## 4. Discussion

In this study, we successfully engineered a recombinant *Lactococcus* sp. KTH0-1S strain capable of whole-cell β-galactosidase activity and demonstrated its application as a whole-cell biocatalyst for lactosucrose synthesis. Compared with conventional intracellular enzyme production systems, this whole-cell biocatalysis approach offers several advantages, most notably the elimination of cell disruption, removal of cell debris, and enzyme purification steps. Instead, intact cells can be directly harvested and reused as biocatalysts, substantially reducing processing time and cost. This advantage is particularly important for large-scale and food-related applications, where downstream processing often represents a major proportion of overall production expenses. Nevertheless, this protein localization requires more sophisticated genetic design than intracellular expression, as it involves both efficient secretion of the target protein and stable anchoring to the cell wall. In this work, the expression construct was rationally designed using the USP45 signal peptide, which is well established for efficient protein secretion in *Lactococcus lactis* [26,27], together with a LysM anchoring domain that mediates non-covalent binding to peptidoglycan [28,29]. *In silico* localization prediction supported the feasibility of this design [30]., and experimental validation confirmed successful localization, with the engineered strain exhibiting β-galactosidase activity of up to 146 Miller Units and the ability to synthesize lactosucrose. These results demonstrate that the USP45–LysM system is an effective strategy for construction of whole-cell biocatalysis in *Lactococcus* sp. KTH0-1S.

Initial attempts to confirm localization of the whole-cell recombinant β-galactosidase by SDS-PAGE and Western blot did not yield a clear, unambiguous band, likely due to the low abundance of the target protein relative to total extracted protein. Dot blot analysis was therefore used as a more sensitive complementary approach, since it eliminates the additional dilution and transfer losses inherent to gel electrophoresis and membrane transfer. Using this approach, a clear signal was detected only in the induced recombinant sample, supporting successful nisin-induced of the His-tagged β-galactosidase. As dot blot is a qualitative rather than quantitative method, this result should be interpreted as confirmatory evidence of protein presence rather than a measure of relative expression level.

Interestingly, whole-genome sequencing revealed that the β-galactosidase from *Lactococcus* sp. KTH0-1S shares only approximately 50% sequence identity with previously reported β-galactosidases. Despite this relatively low sequence similarity, structural modeling indicated a high degree of structural conservation with *Escherichia coli* β-galactosidase (PDB: 1JYX), including preservation of key catalytic residues and cofactor-binding sites. This finding suggests that functional conservation can be maintained despite sequence divergence and provides a structural basis for future protein engineering aimed at enhancing catalytic efficiency, stability, or substrate specificity. The observed variation in β-galactosidase activity among recombinant clones harboring the same expression construct is consistent with well-documented heterogeneity in plasmid copy number [31], secretion efficiency, and surface anchoring capacity in lactic acid bacteria. In addition, stochastic fluctuations in gene expression and physiological differences between individual clones can further contribute to variability in enzyme display levels [32]. Such clone-to-clone variation has been frequently reported in whole-cell systems and underscores the importance of screening multiple clones to identify high-performing strains for downstream applications. It should be noted that only two recombinant clones were characterized in the present study, which represents a limitation. Future work should therefore include broader screening more recombinant colonies to better capture the full range of expression variability and to identify clones with consistently optimal whole-cell biocatalysis efficiency. Induction time was identified as another critical factor influencing whole-cell enzyme activity. β-Galactosidase activity increased during the first 4 h of nisin induction, reflecting efficient protein synthesis, secretion, and anchoring. However, prolonged induction resulted in a decline in activity after 5 h, coinciding with acidification of the culture medium due to lactic acid accumulation. At lower pH values, enzyme stability decreases, and the non-covalent interaction between the LysM domain and peptidoglycan may be weakened, leading to partial enzyme detachment from the cell surface [33]. In addition, prolonged induction from 5 h may affected the enzyme activity due to lower pH from lactic acid formation to 5.28, which close to the isoelectric point of the β-galactosidase (pI = 5.0).

Biochemical characterization of the whole-cell β-galactosidase demonstrated that both pH and temperature strongly influence lactosucrose synthesis. Optimal activity was observed at pH 6.0, whereas markedly reduced activity occurred at pH 5.0, consistent with the predicted isoelectric point (pI = 5.0) of the enzyme, where aggregation or precipitation is more likely to occur [21]. Temperature optimization identified 50 °C as the most favorable condition for lactosucrose production, balancing enhanced reaction kinetics with enzyme stability. At 60 °C, enzyme activity declined significantly, indicating thermal destabilization under prolonged exposure to elevated temperature.

Substrate molar ratio was a decisive parameter affecting lactosucrose synthesis. The highest lactosucrose yield (15,940 mg/L) was achieved at a lactose:sucrose molar ratio of 1:3, indicating that sucrose enrichment strongly favors transgalactosylation. This behavior reflects the dual catalytic functions of β-galactosidase, in which lactose acts as the galactosyl donor and sucrose serves as the acceptor molecule. Excess lactose promotes hydrolysis, whereas excess sucrose increases the likelihood that the galactosyl–enzyme intermediate reacts with an acceptor rather than water, thereby enhancing lactosucrose formation. The optimal ratio observed in this study differs from previous reports using *Bacillus circulans* β-galactosidase [14], suggesting that enzyme origin and active-site architecture play important roles in determining reaction preference.

Molecular docking analysis provided structural support for these biochemical observations. The docking results indicate that Pocket 3 is the energetically most favorable binding site for lactose, sucrose, and galactose, as evidenced by the lowest binding energies obtained among all predicted pockets. The comparable Vina scores observed for sucrose (−7.1 kcal/mol) and galactose (−7.1 kcal/mol), together with the slightly weaker but still favorable binding of lactose (−6.8 kcal/mol), suggest that Pocket 3 can accommodate structurally diverse substrates involved in both hydrolytic and transgalactosylation reactions. Importantly, the strong binding of **sucrose**, which functions as the galactosyl acceptor, supports the experimentally observed enhancement of lactosucrose synthesis under sucrose-enriched conditions. Favorable sucrose binding at Pocket 3 may increase the probability that the galactosyl–enzyme intermediate encounters an acceptor molecule rather than water, thereby shifting the reaction equilibrium toward transgalactosylation. The equally strong interaction observed for **galactose**, an intermediate and product of lactose hydrolysis, suggests that Pocket 3 may also participate in stabilizing reaction intermediates during catalysis. In contrast to Pocket 1, which exhibited moderate binding energies (sucrose −3.8, lactose −3.6, galactose −4.6) and a conserved but less energetically favorable interaction profile, Pocket 3 appears to function as a **high-affinity substrate recognition site**. This distinction may explain why increased sucrose concentration markedly improved lactosucrose yield in the whole-cell system, as strong acceptor binding at Pocket 3 would promote productive enzyme–substrate complexes required for lactosucrose formation.

Despite its high initial activity, the whole-cell biocatalyst exhibited limited reusability, with lactosucrose production decreasing to 10–20% in the second reaction cycle and becoming undetectable in the third cycle. This loss of activity is most likely attributable to the non-covalent nature of LysM-mediated anchoring, which may be destabilized by shear stress, pH fluctuations, or ionic changes during repeated reactions [34]. The complete loss of activity by the third cycle can be explained by the cumulative effect of two main factors: (1) mechanical shear stress during repeated centrifugation and washing steps between cycles, which progressively dislodges the surface-anchored enzyme from the cell wall; and (2) prolonged thermal exposure at 50 °C, which may gradually reduce the binding affinity of the LysM domain to peptidoglycan and promote partial unfolding of the anchoring domain over successive reaction cycles. Collectively, these factors deplete the enzyme pool to levels insufficient for detectable product formation by the third cycle. While LysM anchoring offers simplicity and food-grade compatibility, future studies could focus on improving surface stability through covalent anchoring strategies, multivalent LysM repeats, or protein engineering approaches to enhance binding strength and operational robustness. Additionally, secretion efficiency of the fusion protein may be further enhanced through the incorporation of secretion propeptides together with the USP45 signal peptide [35], which have been shown to improve protein translocation in lactic acid bacteria. Alternative non-covalent cell wall anchoring domains beyond LysM, such as the SH3_5-based CAD4a domain derived from *Lactobacillus plantarum*, may also be explored to achieve more robust surface display on LAB [36].

Collectively, this study demonstrates the successful development of recombinant *Lactococcus* sp. KTH0-1S as a whole-cell β-galactosidase platform and provides comprehensive insights into its catalytic performance, optimal reaction conditions, and current limitations. These findings highlight the potential of GRAS lactic acid bacteria as whole-cell biocatalysts for prebiotic production and establish a solid foundation for further optimization toward industrial implementation.

## 5. Conclusion

This study demonstrates the successful development of a recombinant *Lactococcus* sp. KTH0-1S system displaying whole-cell β-galactosidase activity for efficient lactosucrose synthesis. This strategy enabled the use of whole cells as biocatalysts, eliminating enzyme purification steps and simplifying downstream processing. Optimization of induction conditions, pH, temperature, and substrate molar ratio resulted in high lactosucrose production, with a maximum yield of 15,940 mg/L under sucrose-enriched conditions. Structural modeling and molecular docking provided mechanistic insight into substrate binding and transgalactosylation, supporting the observed biochemical behavior. Although limited reusability was observed due to the non-covalent nature of LysM anchoring, the GRAS status of *Lactococcus* sp. and the demonstrated catalytic performance highlight the potential of this platform for cost-effective and safe prebiotic production. Further optimization of surface anchoring stability is expected to enhance its applicability in industrial bioprocesses.

## Supporting information

S1 Table Primers designed for connecting *SP*, *LacZ*, and *CWA* genes using Gibson Assembly. (DOCX)

S2 Table Comparison of the similarity of β-galactosidase from *Lactococcus* sp. KTH0-1S with database. (DOCX)

S1 File Nucleotide sequence of the expression cassette for surface display of β-galactosidase in *Lactococcus* sp. KTH0-1S. The cassette (3,648 bp) encodes an N-terminal USP45 signal peptide (positions 1–81), His-tag and DDDK enterokinase cleavage site (positions 82–161), β-galactosidase lacZ gene (positions 162–3,078), and C-terminal LysM anchoring domain (positions 3,079–3,648).
(DOCX)

S1 Fig (A) Time-course analysis of ONPG hydrolysis by crude intracellular β-galactosidase from wild-type Lactococcus sp. KTH0-1S at 10, 30, 60, and 240 min. (B) Standard curve for β-galactosidase activity generated using commercial enzyme (0.25–10 U/mL).
(DOCX)

S2 Fig Colony PCR to confirm the presence of USP45-b-galactosidase-CWA (3,648 bp) in *Lactococcus* sp. KTH0-1S. Lane M; 1 kb plus DNA ladder, Lane 1-11; USP45-b-galactosidase-CWA clone 1-11, Lane 12; Negative control.
(DOCX)

S3 Fig HPLC chromatogram profiles confirming Lactosucrose production. The green line represents the standard mixture of each sugars at 0.8% (w/v). The purple line indicates the reaction sample after enzymatic conversion. Sample: 0.3 M Lactose : 0.9 M Sucrose (1:3).
(DOCX)

S4 Fig Graphical abstract. (TIFF)
