# Supplementary material for: β-Galactosidase expression on Lactococcus sp. KTH0-1S as a whole-cell biocatalyst for efficient lactosucrose production
Source: PLoS One. 2026 Sep 25;21(9):e0359091. doi: 10.1371/journal.pone.0359091 (PMC13614602; doi:10.1371/journal.pone.0359091)
Supplement: Supplementary file 1 [file pone.0359091.s005.docx]

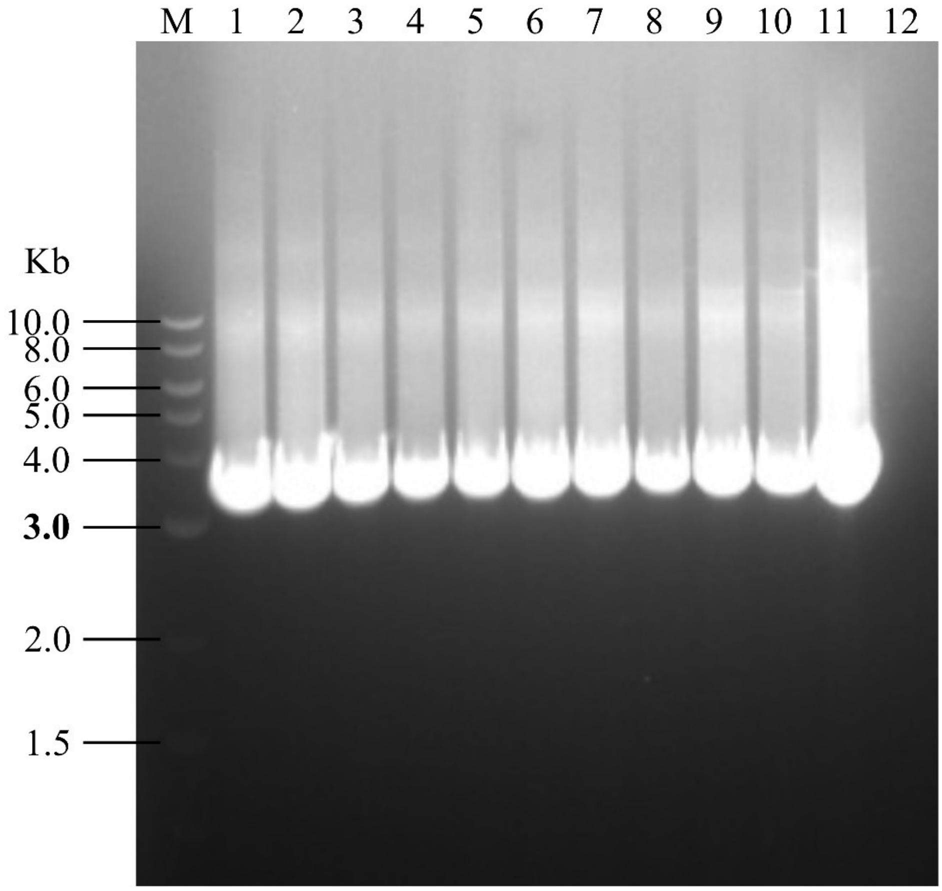
M 1 2 3 4 5 6 7 8 9 10 11 12
Kb
10.0
8.0
6.0
5.0
4.0
3.0
2.0
1.5
