# Supplementary material for: β-Galactosidase expression on Lactococcus sp. KTH0-1S as a whole-cell biocatalyst for efficient lactosucrose production
Source: PLoS One. 2026 Sep 25;21(9):e0359091. doi: 10.1371/journal.pone.0359091 (PMC13614602; doi:10.1371/journal.pone.0359091)
Supplement: Supplementary file 2 [file pone.0359091.s006.docx]

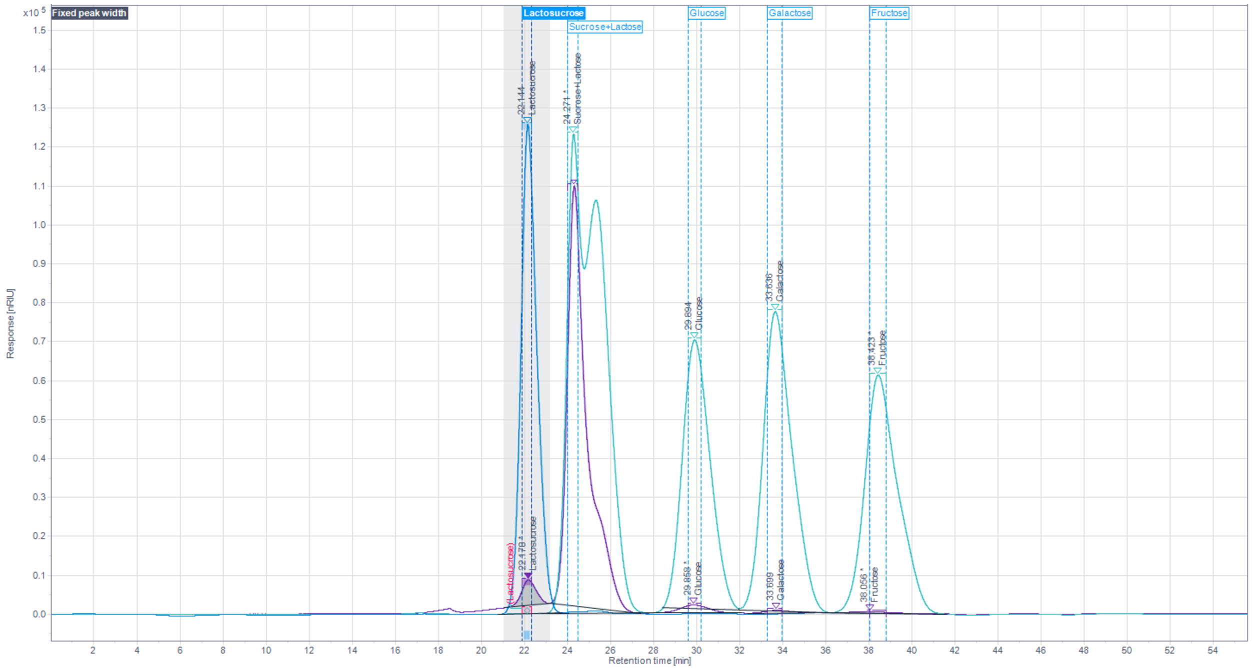

x10 5
Fixed peak width
Lactosucrose
Sucrose+Lactose
Glucose
Galactose
Fructose
22.144
Lactosucrose
24.271 *
Sucrose+Lactose
29.894
Glucose
33.636
Galactose
38.423 *
Fructose
(Lactosucrose)
22.178 *
Lactosucrose
29.858 *
Glucose
33.699
Galactose
38.056 *
Fructose
Response [nRIU]
Retention time [min]
