# Supplementary material for: β-Galactosidase expression on Lactococcus sp. KTH0-1S as a whole-cell biocatalyst for efficient lactosucrose production
Source: PLoS One. 2026 Sep 25;21(9):e0359091. doi: 10.1371/journal.pone.0359091 (PMC13614602; doi:10.1371/journal.pone.0359091)
Supplement: Supplementary file 1 [file pone.0359091.s001.docx]

**Supplementary table1**

| Gene | Primers | Nucleotides |
| --- | --- | --- |
| USP45 | SP F | TTATATTACAGCTCCAGATCGACCTAGTCTTATAACTATACTGACA |
|  | SP R | ATCATTGTCATCATAGCCATCTTATCGTCATCGTCATGATG |
| LacZ | LACZ F | ATCATGACGATGACGATAAGATGGCTATGATGACAATGATTG |
|  | LACZ R | GTGGTTGTCGAGCCACCAGAAGTACTTCCCGTTAATTTAA |
| Anchoring domain | CWA F | TTAAATTAACGGGAAGTACTTCTGGTGGCTCGACAACCAC |
|  | CWA R | GTTCAAAGAAAGCTTGAGCTTTATTTTATTCGTAGATACTGACCAATTAAAATAGT |
