# Supplementary material for: β-Galactosidase expression on Lactococcus sp. KTH0-1S as a whole-cell biocatalyst for efficient lactosucrose production
Source: PLoS One. 2026 Sep 25;21(9):e0359091. doi: 10.1371/journal.pone.0359091 (PMC13614602; doi:10.1371/journal.pone.0359091)
Supplement: Supplementary file 2 [file pone.0359091.s002.docx]

**Supplementary table 2**

| **Sequence** | [**Description**](https://blast.ncbi.nlm.nih.gov/Blast.cgi) | [**Max Score**](https://blast.ncbi.nlm.nih.gov/Blast.cgi?CMD=Get&ADV_VIEW=yes&ADV_VIEW=on&ALIGNMENTS=100&ALIGNMENT_VIEW=Pairwise&BLAST_SPEC=blast2seq&CONFIG_DESCR=ClustMemNbr,ClustComn,Ds,Sc,Ms,Ts,Cov,Eval,Idnt,AccLen,Acc&DATABASE_SORT=0&DESCRIPTIONS=100&DYNAMIC_FORMAT=on&FORMAT_OBJECT=Alignment&FORMAT_TYPE=HTML&GET_SEQUENCE=yes&I_THRESH=&LINE_LENGTH=60&MASK_CHAR=2&MASK_COLOR=1&NEW_VIEW=yes&NUM_OVERVIEW=100&PAGE=Proteins&QUERY_INDEX=0&QUERY_NUMBER=0&RID=7WR4PSE5114&SHOW_LINKOUT=yes&SHOW_OVERVIEW=yes&STEP_NUMBER=&WORD_SIZE=3&ADV_VIEW=on&DISPLAY_SORT=1&HSP_SORT=1) | [**Total Score**](https://blast.ncbi.nlm.nih.gov/Blast.cgi?CMD=Get&ADV_VIEW=yes&ADV_VIEW=on&ALIGNMENTS=100&ALIGNMENT_VIEW=Pairwise&BLAST_SPEC=blast2seq&CONFIG_DESCR=ClustMemNbr,ClustComn,Ds,Sc,Ms,Ts,Cov,Eval,Idnt,AccLen,Acc&DATABASE_SORT=0&DESCRIPTIONS=100&DYNAMIC_FORMAT=on&FORMAT_OBJECT=Alignment&FORMAT_TYPE=HTML&GET_SEQUENCE=yes&I_THRESH=&LINE_LENGTH=60&MASK_CHAR=2&MASK_COLOR=1&NEW_VIEW=yes&NUM_OVERVIEW=100&PAGE=Proteins&QUERY_INDEX=0&QUERY_NUMBER=0&RID=7WR4PSE5114&SHOW_LINKOUT=yes&SHOW_OVERVIEW=yes&STEP_NUMBER=&WORD_SIZE=3&ADV_VIEW=on&DISPLAY_SORT=2&HSP_SORT=1) | [**Query Cover**](https://blast.ncbi.nlm.nih.gov/Blast.cgi?CMD=Get&ADV_VIEW=yes&ADV_VIEW=on&ALIGNMENTS=100&ALIGNMENT_VIEW=Pairwise&BLAST_SPEC=blast2seq&CONFIG_DESCR=ClustMemNbr,ClustComn,Ds,Sc,Ms,Ts,Cov,Eval,Idnt,AccLen,Acc&DATABASE_SORT=0&DESCRIPTIONS=100&DYNAMIC_FORMAT=on&FORMAT_OBJECT=Alignment&FORMAT_TYPE=HTML&GET_SEQUENCE=yes&I_THRESH=&LINE_LENGTH=60&MASK_CHAR=2&MASK_COLOR=1&NEW_VIEW=yes&NUM_OVERVIEW=100&PAGE=Proteins&QUERY_INDEX=0&QUERY_NUMBER=0&RID=7WR4PSE5114&SHOW_LINKOUT=yes&SHOW_OVERVIEW=yes&STEP_NUMBER=&WORD_SIZE=3&ADV_VIEW=on&DISPLAY_SORT=4&HSP_SORT=0) | [**E value**](https://blast.ncbi.nlm.nih.gov/Blast.cgi?CMD=Get&ADV_VIEW=yes&ADV_VIEW=on&ALIGNMENTS=100&ALIGNMENT_VIEW=Pairwise&BLAST_SPEC=blast2seq&CONFIG_DESCR=ClustMemNbr,ClustComn,Ds,Sc,Ms,Ts,Cov,Eval,Idnt,AccLen,Acc&DATABASE_SORT=0&DESCRIPTIONS=100&DYNAMIC_FORMAT=on&FORMAT_OBJECT=Alignment&FORMAT_TYPE=HTML&GET_SEQUENCE=yes&I_THRESH=&LINE_LENGTH=60&MASK_CHAR=2&MASK_COLOR=1&NEW_VIEW=yes&NUM_OVERVIEW=100&PAGE=Proteins&QUERY_INDEX=0&QUERY_NUMBER=0&RID=7WR4PSE5114&SHOW_LINKOUT=yes&SHOW_OVERVIEW=yes&STEP_NUMBER=&WORD_SIZE=3&ADV_VIEW=on&DISPLAY_SORT=0&HSP_SORT=0) | [**% Ident**](https://blast.ncbi.nlm.nih.gov/Blast.cgi?CMD=Get&ADV_VIEW=yes&ADV_VIEW=on&ALIGNMENTS=100&ALIGNMENT_VIEW=Pairwise&BLAST_SPEC=blast2seq&CONFIG_DESCR=ClustMemNbr,ClustComn,Ds,Sc,Ms,Ts,Cov,Eval,Idnt,AccLen,Acc&DATABASE_SORT=0&DESCRIPTIONS=100&DYNAMIC_FORMAT=on&FORMAT_OBJECT=Alignment&FORMAT_TYPE=HTML&GET_SEQUENCE=yes&I_THRESH=&LINE_LENGTH=60&MASK_CHAR=2&MASK_COLOR=1&NEW_VIEW=yes&NUM_OVERVIEW=100&PAGE=Proteins&QUERY_INDEX=0&QUERY_NUMBER=0&RID=7WR4PSE5114&SHOW_LINKOUT=yes&SHOW_OVERVIEW=yes&STEP_NUMBER=&WORD_SIZE=3&ADV_VIEW=on&DISPLAY_SORT=3&HSP_SORT=3) | [**Acc. Len**](https://blast.ncbi.nlm.nih.gov/Blast.cgi) |
| --- | --- | --- | --- | --- | --- | --- | --- |
| *L. lactis* KTH0-1S β-Galactosidase | [1JYX_1\|Chains A, B, C, D\|Beta-Galactosidase\|Escherichia coli (562)](https://blast.ncbi.nlm.nih.gov/Blast.cgi) | 965 | 965 | 98% | 0.0 | 50.39% | 1023 |
