# Supplementary material for: β-Galactosidase expression on Lactococcus sp. KTH0-1S as a whole-cell biocatalyst for efficient lactosucrose production
Source: PLoS One. 2026 Sep 25;21(9):e0359091. doi: 10.1371/journal.pone.0359091 (PMC13614602; doi:10.1371/journal.pone.0359091)
Supplement: Supplementary file 3 [file pone.0359091.s003.docx]

**S1 Fig.** Nucleotide sequence of the codon-optimized expression cassette for *Lactococcus* sp. KTH0-1S.
