# Supplementary material for: β-Galactosidase expression on Lactococcus sp. KTH0-1S as a whole-cell biocatalyst for efficient lactosucrose production
Source: PLoS One. 2026 Sep 25;21(9):e0359091. doi: 10.1371/journal.pone.0359091 (PMC13614602; doi:10.1371/journal.pone.0359091)
Supplement: Supplementary file 7 [file pone.0359091.s008.docx]

**Response to Reviewer 2**

We sincerely thank both reviewers for their thorough and constructive comments. Below is our point-by-point response. Text added or revised in the manuscript is highlighted in yellow in the revised document.

***Comment 1:*** This paper did not appear to provide the sequence of this specific *Lactococcus* sp. KTH0-1S β-galactosidase. Please include this information. How is the homology vs other Lactococcus species in databases?

***Answer 1:*** We thank the reviewer for this important observation. BLAST analysis against the NCBI non-redundant protein database restricted to the genus Lactococcus revealed highest sequence identity to β-galactosidase from L. lactis (97.19%, 100% query coverage; WP_404378437.1), followed by L. kimchii (69.32%), L. taiwanensis (64.04%), L. hircilactis (58.79%), and L. termiticola (55.69%), confirming broad conservation of this GH2 enzyme within the Lactococcus genus. The manuscripts has been revised in the methods Sections 2.8 and this result has been added as suggested in Section 3.3 (line 294-300).

***Comment 2:*** The reference source for L. sp. KTH0-1S is mentioned as 'this study'. But I believe the same strain was isolated and characterized earlier.

***Answer 2:*** Thank you for the comment. The Table 1 has been updated to cite Reference 19 (Saelao et al., Food Control, 2018) as the original source of the strain as suggested.

***Comment 3:*** In 2.2.3, a final 25% 1:1 lactose:sucrose was used. However, in subsequent experiments (e.g., 2.4), 12.5% solutions were used. Please explain rationale.

***Answer 3:*** The 25% (w/v) substrate concentration in Section 2.2.3 was used for initial validation of lactosucrose synthesis capability with crude enzyme extract. Based on those results, residual substrate remained in excess at the end of the reaction, indicating the reaction was not substrate-limited. Therefore, 12.5% (w/v) was selected for whole-cell experiments in later experiments to minimize osmotic stress on intact cells and to facilitate substrate dissolution, while still providing sufficient substrate for product formation. However the first experiment with 25% substrate were conducted under different system conditions (crude enzyme from WT vs. whole cells recombinant) and were not intended for direct comparison.

***Comment 4:*** Please check and ensure that 'In silico' should be in italics (e.g., 2.8, line 289, 483).

***Answer 4:*** All occurrences of 'in silico' throughout the manuscript (Section 2.8 heading, 3.4 body text and discussion) have been formatted in italics in the revised document.

***Comment 5:*** Table 4: Why pick only 2 clones since the differences are significant.

***Answer 5:*** We thank the reviewer for this valid comment. We acknowledge that screening only two clones is a limitation of this study. The two clones were selected based on positive ONPG activity and served as representative examples to demonstrate the feasibility of the surface display system. We agree that a broader clone screening would better capture expression variability. This limitation has been discussed in the Discussion section (line 541-545) of the revised manuscript, and future work will include screening of a larger number of clones to identify variants with optimal surface display efficiency.

***Comment 6:*** Fig 3 caption. Please define the 'identical' conditions used.

***Answer 6:*** The Figure 3 caption has been revised to explicitly define the conditions as suggested to avoid confusion: recombinant *Lactococcus* sp. KTH0-1S cells at OD₆₀₀ = 20; lactose and sucrose each at 12.5% (w/v); 50 °C; 4 h incubation. (line 392-393)

***Comment 7:*** 3:1 (and 1:3) lactose:sucrose ratios gave notable higher yields. Why not go higher (e.g., 4:1 or 1:4)?

***Answer 7:*** This is an interesting suggestion. The molar ratios tested were constrained by substrate solubility at the concentrations used; higher concentration (4:1 or 1:4) would require one substrate at a concentration approaching its solubility limit, potentially causing precipitation and inconsistent results.

***Comment 8:*** Secretion of USP45 tagged proteins can also be included by use of secretion propeptides and anchoring of the enzyme can be attempted with other cell anchoring domains.

***Answer 8:*** We thank the reviewer for these valuable suggestions. As recommended, we have expanded the future perspectives section in the Discussion to include: the use of different secretion propeptides in combination with the USP45 signal peptide to enhance secretion efficiency and the exploration of alternative non-covalent cell wall anchoring domains, such as the SH3_5-based CAD4a domain, as alternatives to LysM for potentially more robust surface display on LAB have been added to the revised manuscript (line 608-613)
