# Supplementary material for: β-Galactosidase expression on Lactococcus sp. KTH0-1S as a whole-cell biocatalyst for efficient lactosucrose production
Source: PLoS One. 2026 Sep 25;21(9):e0359091. doi: 10.1371/journal.pone.0359091 (PMC13614602; doi:10.1371/journal.pone.0359091)
Supplement: Supplementary file 8 [file pone.0359091.s009.docx]

**Response to Reviewer 1 (Round 2)**

We thank the Editor and Reviewer for their continued attention to this important point. Below is our point-by-point response. Text added or revised in the manuscript is highlighted in yellow in the revised document.

***Comment 1:*** *“I still want to see a direct proof that the enzyme is in the surface... A simple western blot would be sufficient but it would definitely proof the presence of the target enzyme in the surface and successfully expression in that location.” (Reviewer 1)*

*“I think either you need to proof the localization of the target protein e.g. by means of a western plot or simplify the message.” (Editor)*

***Answer 1:*** We thank the reviewer and editor for this comment. We attempted Western blot analysis of the LiCl-extracted cell surface fraction using an anti-His antibody; however, SDS-PAGE and Western blot did not yield a clear, unambiguous band, likely due to the low abundance of the surface-displayed protein relative to the total extracted protein.

To provide direct protein-level evidence, we therefore performed dot blot analysis, a more sensitive immunodetection approach that avoids the additional dilution and transfer losses inherent to gel electrophoresis and membrane transfer. LiCl-extracted surface protein fractions from wild-type and recombinant *Lactococcus* sp. KTH0-1S, under both non-induced and nisin-induced conditions, were probed with anti-His antibody. A distinct immunoreactive signal was detected exclusively in the induced recombinant sample, with no signal observed in the non-induced recombinant sample or in either wild-type sample (Fig 8). This result provides direct immunological evidence that the His-tagged β-galactosidase is expressed and displayed on the cell surface, dependent on nisin induction, and complements the functional and genetic evidence presented in our previous response.

This new result and its methodology have been added to the revised manuscript (Method 2.4.3 (line 184-225), Result 3.11 (line 551-566) and Fig 8, with interpretation provided in the Discussion (line 588-596).
