# Supplementary material for: β-Galactosidase expression on Lactococcus sp. KTH0-1S as a whole-cell biocatalyst for efficient lactosucrose production
Source: PLoS One. 2026 Sep 25;21(9):e0359091. doi: 10.1371/journal.pone.0359091 (PMC13614602; doi:10.1371/journal.pone.0359091)
Supplement: Supplementary file 9 [file pone.0359091.s010.docx]

**Response to Editor/Reviewer**

Manuscript ID: **PONE-D-26-01975R2**

**Editor Comments** : *Please show either evidence for the enzyme being located in the surface as stated or rephrase the statement.*

**Response:**

We thank the Editor for this observation and have chosen to rephrase the statement throughout the manuscript. Specifically, we have replaced or removed all references to “surface display” with “expression” (or equivalent, more conservative wording such as “whole-cell biocatalyst/ whole-cell recombinant” where appropriate) throughout the manuscript. This revision has been applied consistently to:

– The manuscript title, which now “β-Galactosidase Expression on *Lactococcus* sp. KTH0-1S as a Whole-Cell Biocatalyst for Efficient Lactosucrose Production” (previously “Surface Display of β-Galactosidase on *Lactococcus* sp. KTH0-1S as a Whole-Cell Biocatalyst for Efficient Lactosucrose Production”).

– The Abstract.

Line 31 exhibited a maximum whole-cell β-galactosidase activity

Line 32 The whole-cell enzyme efficiently catalyzed lactosucrose synthesis

– The Introduction

Line 121 This work demonstrates the feasibility of employing a GRAS Lactococcus host as a safe and efficient whole-cell biocatalysis platform for cost-effective lactosucrose production

– Material and methods

Line 170 Construction of whole-cell recombinant *Lactococcus* sp. displaying β-galactosidase

Line 186 The whole-cell β-galactosidase activity was measured using ONPG

Line 218 To compare ~~surface~~ protein expression between *Lactococcus* sp.

Line 251-252 using whole-cell biocatalyst recombinant Lactococcus sp. with β-galactosidase (final OD₆₀₀ = 100).

- The Results sections

Line 340 Design of the β-galactosidase expression construct for whole-cell immobilized Lactococcus sp.

Line 342 to enable whole-cell biocatalysis of β-galactosidase in Lactococcus sp. KTH0-1S.

Line 364 Prediction of ~~surface displayed~~ protein localization

Line 382 Screening of recombinant Lactococcus sp. clones for whole-cell immobilized β-galactosidase activity

Line 383 Recombinant Lactococcus sp. KTH0-1S clones harboring the expression construct

Line 388 No detectable β-galactosidase activity was observed using whole-cell of the wild-type

Line 402 both recombinant clones exhibited a marked increase in whole-cell β-galactosidase activity compared with the corresponding non-induced controls

Line 411 Whole-cell β-galactosidase activity of recombinant Lactococcus sp. KTH0-1S clones under induced and non-induced conditions

Line 418 Effect of induction time on the activity of whole-cell β-galactosidase in recombinant Lactococcus sp.

Line 420 The effect of induction time on the activity of whole-cell β-galactosidase

Line 424 Whole-cell β-galactosidase activity increased progressively during the early induction period

Line 431 Effect of induction time on whole-cell β-galactosidase activity and culture pH

Line 439 The effect of pH on the catalytic activity of whole-cell β-galactosidase from recombinant Lactococcus sp. KTH0-1S

Line 456 lactosucrose synthesis using whole-cell β-galactosidase in Lactococcus sp. lies between pH 6.0 and 8.0.

Line 459 Effect of reaction pH on lactosucrose synthesis catalyzed by whole-cell β-galactosidase in recombinant Lactococcus sp.

Line 468-469 The effect of temperature on lactosucrose synthesis catalyzed by whole-cell β-galactosidase in recombinant Lactococcus sp.

Line 478-479 Effect of temperature and reaction time on lactosucrose production catalyzed by whole-cell β-galactosidase in recombinant Lactococcus sp. KTH0-1S.

Line 490-491 the optimal temperature for lactosucrose synthesis using whole-cell β-galactosidase under the tested conditions.

Line 494-495 The molar ratio of lactose to sucrose significantly affected lactosucrose synthesis catalyzed by whole-cell β-galactosidase in recombinant Lactococcus sp. KTH0-1S

Line 511-512 Effect of lactose:sucrose molar ratio on lactosucrose production and reusability of whole-cell β-galactosidase in recombinant Lactococcus sp. KTH0-1S.

Line 585 Confirmation of whole-cell β-galactosidase expression by dot blot

Line 591-592 These results confirm that the His-tagged β-galactosidase was successfully expressed of the recombinant *Lactococcus* sp. KTH0-1S

Line 597 Dot blot analysis of whole-cell β-galactosidase in Lactococcus sp. KTH0-1S.

- – The Discussion

Line 602-603 Lactococcus sp. KTH0-1S strain capable of whole-cell β-galactosidase activity

Line 613-614 this whole-cell biocatalysis approach offers several advantages, most notably the elimination of cell disruption,

Line 618-619 Nevertheless, this protein localization requires more sophisticated genetic design than intracellular expression

Line 624-625 experimental validation confirmed successful localization, with the engineered strain exhibiting β-galactosidase activity

Line 627 construction of whole-cell biocatalysis in Lactococcus sp. KTH0-1S.

Line 660-663 Future work should therefore include broader screening more recombinant colonies to better capture the full range of expression variability and to identify clones with consistently optimal whole-cell biocatalysis efficiency. Induction time was identified as another critical factor influencing whole-cell enzyme activity.

Line 673 Biochemical characterization of the whole-cell β-galactosidase demonstrated that both pH and temperature

Line 713 Despite its high initial activity, the ~~surface-displayed~~ whole-cell biocatalyst exhibited limited reusability

Line 737 as a whole-cell β-galactosidase platform

- Conclusion

Line 744-745 This study demonstrates the successful development of a recombinant Lactococcus sp. KTH0-1S system displaying whole-cell β-galactosidase activity for efficient lactosucrose synthesis.
