# Supplementary material for: β-Galactosidase expression on Lactococcus sp. KTH0-1S as a whole-cell biocatalyst for efficient lactosucrose production
Source: PLoS One. 2026 Sep 25;21(9):e0359091. doi: 10.1371/journal.pone.0359091 (PMC13614602; doi:10.1371/journal.pone.0359091)
Supplement: Peer review history

# Peer review: Decision Letter 0

**DOI:** 10.1371/journal.pone.0359091.r001

**Authors:** Dirk Tischler

21 Mar 2026

Dear Dr. Watthanasakphuban,

Thank you for submitting your manuscript to PLOS ONE. After careful consideration, we feel that it has merit but does not fully meet PLOS ONE’s publication criteria as it currently stands. Therefore, we invite you to submit a revised version of the manuscript that addresses the points raised during the review process.

Please submit your revised manuscript by May 05 2026 11:59PM. If you will need more time than this to complete your revisions, please reply to this message or contact the journal office at plosone@plos.org. When you're ready to submit your revision, log on to https://www.editorialmanager.com/pone/ and select the 'Submissions Needing Revision' folder to locate your manuscript file.

- A letter that responds to each point raised by the academic editor and reviewer(s). You should upload this letter as a separate file labeled 'Response to Reviewers'.
- A marked-up copy of your manuscript that highlights changes made to the original version. You should upload this as a separate file labeled 'Revised Manuscript with Track Changes'.
- An unmarked version of your revised paper without tracked changes. You should upload this as a separate file labeled 'Manuscript'.

If you would like to make changes to your financial disclosure, please include your updated statement in your cover letter. Guidelines for resubmitting your figure files are available below the reviewer comments at the end of this letter.

If applicable, we recommend that you deposit your laboratory protocols in protocols.io to enhance the reproducibility of your results. Protocols.io assigns your protocol its own identifier (DOI) so that it can be cited independently in the future. For instructions see: https://journals.plos.org/plosone/s/submission-guidelines#loc-laboratory-protocols. Additionally, PLOS ONE offers an option for publishing peer-reviewed Lab Protocol articles, which describe protocols hosted on protocols.io. Read more information on sharing protocols at https://plos.org/protocols?utm_medium=editorial-email&utm_source=authorletters&utm_campaign=protocols.

We look forward to receiving your revised manuscript.

Kind regards,

Dirk Tischler

Academic Editor

PLOS One

Journal Requirements:

When submitting your revision, we need you to address these additional requirements.

1.Please ensure that your manuscript meets PLOS ONE's style requirements, including those for file naming. The PLOS ONE style templates can be found at

https://journals.plos.org/plosone/s/file?id=wjVg/PLOSOne_formatting_sample_main_body.pdf and

https://journals.plos.org/plosone/s/file?id=ba62/PLOSOne_formatting_sample_title_authors_affiliations.pdf

2. PLOS requires an ORCID iD for the corresponding author in Editorial Manager on papers submitted after December 6th, 2016. Please ensure that you have an ORCID iD and that it is validated in Editorial Manager. To do this, go to ‘Update my Information’ (in the upper left-hand corner of the main menu), and click on the Fetch/Validate link next to the ORCID field. This will take you to the ORCID site and allow you to create a new iD or authenticate a pre-existing iD in Editorial Manager.

3. Thank you for stating the following financial disclosure:

“This research is funded by the Fundamental Fund: Basic Research Fund (FF(KU)16.67) from the Kasetsart University Research and Development Institute (KURDI), Bangkok, Thailand. This research is funded by Kasetsart University through the Graduate School Fellowship Program.”

Please state what role the funders took in the study.  If the funders had no role, please state: "The funders had no role in study design, data collection and analysis, decision to publish, or preparation of the manuscript."

If this statement is not correct you must amend it as needed.

Please include this amended Role of Funder statement in your cover letter; we will change the online submission form on your behalf.

4. Thank you for stating the following in the Acknowledgments Section of your manuscript:

“This research is funded by the Fundamental Fund: Basic Research Fund (FF(KU)16.67) from the Kasetsart University Research and Development Institute (KURDI), Bangkok, Thailand. This research is funded by Kasetsart University through the Graduate School Fellowship Program”

We note that you have provided funding information that is not currently declared in your Funding Statement. However, funding information should not appear in the Acknowledgments section or other areas of your manuscript. We will only publish funding information present in the Funding Statement section of the online submission form.

Please remove any funding-related text from the manuscript and let us know how you would like to update your Funding Statement. Currently, your Funding Statement reads as follows:

“This research is funded by the Fundamental Fund: Basic Research Fund (FF(KU)16.67) from the Kasetsart University Research and Development Institute (KURDI), Bangkok, Thailand. This research is funded by Kasetsart University through the Graduate School Fellowship Program.”

Please include your amended statements within your cover letter; we will change the online submission form on your behalf.

5. If the reviewer comments include a recommendation to cite specific previously published works, please review and evaluate these publications to determine whether they are relevant and should be cited. There is no requirement to cite these works unless the editor has indicated otherwise.

[Note: HTML markup is below. Please do not edit.]

Reviewer's Responses to Questions

**Comments to the Author**

1. Is the manuscript technically sound, and do the data support the conclusions?

Reviewer #1: No

Reviewer #2: Yes

**********

2. Has the statistical analysis been performed appropriately and rigorously?

Reviewer #1: No

Reviewer #2: Yes

**********

3. Have the authors made all data underlying the findings in their manuscript fully available?

The PLOS Data policy

Reviewer #1: No

Reviewer #2: No

**********

4. Is the manuscript presented in an intelligible fashion and written in standard English?

Reviewer #1: Yes

Reviewer #2: Yes

**********

Reviewer #1: Overall, the paper shows the surface display of a galactosidase in Lactococcus sp. KTH0-1S for the production of lactosucrose. I don’t see any mention about what GH family this b-galactosidase is, maybe a GH family 1?. A little. bit of context of where this enzyme comes from would be beneficial for context.

Overall, this study could benefit from more experimental design as I believe that this could enhance the quality of this paper, especially proving the location of the anchored protein into the surface of the bacterium. I suggest obtaining those confirmations before submitting again to another journal. Therefore, I think this paper needs to be massively imporved with those experiments.

Line 57, it would beneifitial to show the chemical structure of lactosucrose for the non-specialist reader.

Line 90, I was trying to read about the LysM anchoring domain in references 18 and 19 but there is nothing or little there. The authours needs to explain better what these domains are as they have not been defined in the introduction at all. In addition, from all Lactococcus strains, why did they pick Lactococcus sp. KTH0-1S strain in particular? I didn’t find the rationale to pick this strain; I might miss anything in the text so please clarify that.

Line 237-238. The lack of linear range in Figure S1A is not due to substrate depletion or product inhibition. Instead, the authors obtained an extremely high absorbance and the phototube in the spectrophotometer didn’t detect the absorbance as it was saturated. I think if they want to quantify properly the rate, they will need to dilute the reaction 1/10 to be in range with the detection limit of the phototube. I am afraid that this would need to be repeated as the estimated enzyme activity could be over calculated.

Line 246-250. I haven’t seen any HPLC data. I would love to see any trace to see how pure the conversion and the efficiency of the transglycosilation is.

Table 2. What is conversion 1 and conversion 2? Replicates?. More detail is required.

Lines 268-276. I can’t see any informationa bout the sequence of the native b-galactosidase from Lactococcus sp. KTH0-1S at all. Authors need to specify the exact gene that they cloned, even the locus tag would be beneficial. Table S1 only contains the primers used for the amplification of the cassette but there is no confimation that the homologous recombination was successful. I suggest an western blot to confirm that the protein is displayed in the surface or just a sequencing of the positive clones by sanger sequencing.

Lines 295-323. The authors assumed that the display worked because they have activity in the surface after the induction, which probably is. However, a confirmation that no other protein is present in the surface (Western blot) would be required to confirm that the protein is present and active after induction.

Line 331-334. The induction time of 5h resulted in a reduction in enzyme activity. Is it possible that the absorbance was too high in the phototube, again?.

Fig. 3 and 4. The authors reported the mean +/- SD of three replicated. Where are the errors in the figures?.

Table 5. The authors didn;t see anya ctivity after cycle 3 and they mentioned that catalytic activity was lost. Any idea why is this? Some discussion should be presented.

Lines 414, as far as I understand AlphaFold3 modelling, it only predicts monomers. Why the authors predicted to be homotetramer? Is there any other comparison they did?. Also, quality of the figures 5-7 is terrible, do they have a high quality figure for the revision?.

Line 442, a magnesium ion was observed in the protein from E. coli, no in the model from AlphaFold. The way that is presented was confusing. An AlphaFold3 model only presents the model of the protein, but it doesn’t incorporate any ion, only X-ray or other lab-based technique, does that.

Reviewer #2: Main comments are on:

1) Lactococcus sp. KTH0-1S is an unique strain isolated from fermented shrimp in Thailand and the strain does not fit into known established strains like L. lactis. Access to this strain will require an MTA from the thai group. While its genome sequence has been published in early 2025, thus paper did not appear to provide the sequence of this specific L. sp. KTH0-1SB-Galacotosidase (although the primer sequences was provided). Please include these information. how is the homology of L. sp. KTH0-1SB-Galacotosidase vs other Lactococcus species in databases?

2) In Table 1, the reference source for L. sp. KTH0-1S is mentioned as this study. But i believe the same strain was isolated and characterized earlier. It is advisable to put in the original source/paper for this strain in Table 1.

Minor:

1) in 2,2,3. a final 25% 1:1 lactose:sucrose was used. However, in subsequent experiments (e.g. 2.4), 12.5% solutions were used. Please explain rationale.

2) please check and ensure that "In silico" should be in italics (e.g. 2.8, line 289, 483).

3) Table 4: Why pick only 2 clones since the differences are significant.

4) Fig 3 caption. Please define the "identical" conditions used.

5) Table 5: 3:1 (and 1:3) lactose:sucrose ratios gave notable higher yields. Why not go higher (e.g. 4:1 or 1:4)?

6) Line 555-558: on future studies: secretion of USP45 tagged proteins can also be included by used of secretion propeptides (e.g. Microb Cell Fact. 2017. 5;16(1):221.) and anchoring of the enzyme can be attempted with other cell anchoring domains (e.g. https://doi.org/10.3389/fbioe.2020.614498_

**********

what does this mean?). If published, this will include your full peer review and any attached files.

If you choose “no”, your identity will remain anonymous but your review may still be made public.

**Do you want your identity to be public for this peer review?**  For information about this choice, including consent withdrawal, please see our Privacy Policy

Reviewer #1: No

Reviewer #2: **Yes:**  Dave Siak-Wei Ow

**********

[NOTE: If reviewer comments were submitted as an attachment file, they will be attached to this email and accessible via the submission site. Please log into your account, locate the manuscript record, and check for the action link "View Attachments". If this link does not appear, there are no attachment files.]

To ensure your figures meet our technical requirements, please review our figure guidelines: https://journals.plos.org/plosone/s/figures

You may also use PLOS’s free figure tool, NAAS, to help you prepare publication quality figures: https://journals.plos.org/plosone/s/figures#loc-tools-for-figure-preparation.

NAAS will assess whether your figures meet our technical requirements by comparing each figure against our figure specifications.

# Author comment: Author response to Decision Letter 1

**DOI:** 10.1371/journal.pone.0359091.r002

8 Jun 2026

Response to Reviewer 1

We sincerely thank both reviewers for their thorough and constructive comments. Below is our point-by-point response. Text added or revised in the manuscript is highlighted in yellow in the revised document.

Comment 1: I don't see any mention about what GH family this β-galactosidase is, maybe a GH family 1?

Answer 1: We thank the reviewer for this important observation. To address this, we performed sequence analysis of the β-galactosidase from Lactococcus sp. KTH0-1S against the CAZy database using the dbCAN2 server (hmmscan). The analysis confirmed that this enzyme belongs to glycoside hydrolase (GH) family 2 (top hit: GH2_10.hmm; E-value = 2×10⁻²⁶⁸; coverage = 98.8%). This classification is consistent with its sequence similarity to Escherichia coli β-galactosidase (PDB: 1JYX), a well-characterized GH2 enzyme. A sentence clarifying the GH family classification has been added to the Introduction (line 74-78).

Comment 2: This study could benefit from more experimental design, especially proving the location of the anchored protein into the surface of the bacterium.

Response: We thank the reviewer for this comment. Although direct protein-level confirmation by Western blot was attempted but technically limited, we respectfully submit that three complementary lines of evidence collectively support successful surface display of β-galactosidase in Lactococcus sp. KTH0-1S.

First, the cell-surface localization mechanism of LysM-containing proteins in lactic acid bacteria is well established in the literature. LysM domains mediate non-covalent binding to N-acetylglucosamine residues in peptidoglycan, and their cell wall localization has been directly demonstrated by immunofluorescence microscopy and cell fractionation in multiple studies, as cited in the manuscript (line 107-109). The well-characterized LysM anchoring mechanism therefore provides strong a priori support for cell surface localization of the fusion protein.

Second, functional evidence directly demonstrates that β-galactosidase was accessible on the cell surface. Intact, non-disrupted whole cells successfully catalyzed lactosucrose synthesis from extracellular substrates — a reaction that would not be possible if the enzyme were located intracellularly, as lactose and sucrose cannot freely cross an intact bacterial membrane. This constitutes direct functional proof of surface-accessible enzyme activity.

Third, β-galactosidase activity was exclusively detected in nisin-induced recombinant cells and was completely absent in both non-induced recombinant cells and wild-type cells under identical assay conditions. Since the induced and non-induced samples were derived from the same recombinant clone, the induction-dependent activity pattern can only be explained by NICE-driven expression and surface display, ruling out any endogenous or non-specific β-galactosidase contribution.

Comment 3: It would be beneficial to show the chemical structure of lactosucrose for the non-specialist reader.

Answer 3: We agree. The chemical structure of lactosucrose [O-β-D-galactopyranosyl-(1,4)-O-α-D-glucopyranosyl-(1,2)-β-D-fructofuranoside] has been added in the graphical abstract.

Comment 4: The authors need to explain better what these domains are as they have not been defined in the introduction at all.

Answer 4: A clear definition of the LysM domain has been added to the Introduction: “LysM (lysin motif) domains are non-catalytic peptidoglycan-binding modules of approximately 40–60 amino acids that mediate non-covalent attachment to N-acetylglucosamine residues in the bacterial cell wall. (line 107-109)

Comment 5: Why did they pick Lactococcus sp. KTH0-1S strain in particular? I didn't find the rationale to pick this strain.

Answer 5: Lactococcus sp. KTH0-1S was selected for the following reasons: (1) It is an indigenous Thai isolate from fermented shrimp (Kung-Som) with a fully sequenced genome [18,19]; (2) whole-genome analysis confirmed the absence of antimicrobial resistance genes and virulence factors, supporting its food-grade suitability; (3) the genome encodes the NisRK two-component regulatory system required for nisin-controlled gene expression (NICE), enabling robust inducible protein production; and (4) genomic and phenotypic evidence confirmed functional respiration metabolism upon heme supplementation, allowing high-cell-density cultivation for enhanced biomass yield. This information has been added to the introduction (line 93-101)

Comment 6: Fig S1A : The lack of linear range in Figure S1A is not due to substrate depletion or product inhibition. Instead, the authors obtained an extremely high absorbance and the phototube in the spectrophotometer didn't detect the absorbance as it was saturated.

Answer 6: We thank the reviewer for this concern. We would like to clarify that the OD₄₂₀ values reported in Section 3.1 and Figure S1A are back-calculated values after correction for the dilution factor applied prior to measurement. Raw absorbance readings were maintained within the linear detection range of the spectrophotometer (OD < 1.0) by diluting the reaction mixture before each measurement. Detector saturation was therefore not a concern. To further confirm this, the experiment was independently repeated and yielded fully consistent results.

Comment 7: I haven't seen any HPLC data. I would love to see any trace to see how pure the conversion and the efficiency of the transglycosylation is.

Answer 7: We thank the reviewer for this suggestion. HPLC chromatogram data have been included as Supplementary Figure S2 in the revised manuscript. The analysis was performed using an Aminex HPX-87C column with refractive index detection. As shown in Fig. S2, all sugars were clearly resolved with distinct retention times: lactosucrose (~22.1 min), sucrose+lactose (~24.3 min), glucose (~29.9 min), galactose (~33.6 min), and fructose (~38.1 min). The reaction sample (0.3 M lactose : 0.9 M sucrose, molar ratio 1:3) showed a distinct product peak co-eluting with the authentic lactosucrose standard, confirming the identity of the product. Furthermore, the chromatogram revealed a corresponding decrease in residual sucrose and lactose peaks relative to the standard mixture, consistent with substrate consumption during enzymatic transglycosylation. A sentence referring to Fig. S2 has been added to Section 3.9 (line 429-434) of the revised manuscript.

Comment 8: Table 2 What is conversion 1 and conversion 2? Replicates? More detail is required.

Answer 8: Conversion 1 and Conversion 2 refer to independent reactions at two different time points (4 h and 4.5 h, respectively), not technical replicates. A clarifying note has been added to the Table 2 legend. (line 284-286)

Comment 9: I can't see any information about the sequence of the native β-galactosidase from Lactococcus sp. KTH0-1S at all. Authors need to specify the exact gene that they cloned, even the locus tag would be beneficial.

Answer 9: We thank the reviewer for these important comments. We would like to address each point as follows.

Regarding the gene sequence, the lacZ gene encoding β-galactosidase was identified at genomic coordinates 2,064,341–2,067,337 of the Lactococcus sp. KTH0-1S genome based on KEGG database analysis, as stated in Section 3.3. The full nucleotide sequence of the expression cassette (3,648 bp), comprising the USP45 signal peptide, lacZ gene and LysM anchoring domain, has been included as Supplementary Information S1 in the revised manuscript.

Regarding confirmation of successful cloning, colony PCR was performed using construct-specific primers. All screened colonies yielded a band of the expected size (~3,648 bp), confirming successful insertion of the expression cassette into pNZ8150 (Fig. S2). Positive clones were further verified by Sanger sequencing, which confirmed the correct sequence of the cloned cassette. A sentence referring to these results has been added to Section 3.3 of the revised manuscript. (line 303-307)

Comment 10: I suggest a western blot to confirm that the protein is displayed in the surface or just a sequencing of the positive clones by Sanger sequencing.

Answer 10: Positive clones were confirmed by colony PCR (insert size 3,648 bp) and Sanger sequencing, as detailed above and has been mentioned in the 2.3.1 as “The recombinant plasmid was propagated in E. coli DH5α by electroporation and verified by colony PCR, agarose gel electrophoresis, and sequencing.”

The sequencing result has been provided in the Supplementary Information S1." And has been mentioned in the manuscript as

Successful insertion of the expression cassette into pNZ8150 was confirmed by colony PCR using construct-specific primers, which yielded a band of the expected size (~3,648 bp) in all screened colonies (Fig. S2). The correct sequence of the cloned cassette was further verified by Sanger sequencing, and the full nucleotide sequence of the USP45–lacZ–LysM expression cassette is provided in Supplementary Information S1." (line 303-307)

Comment 11: The induction time of 5h resulted in a reduction in enzyme activity. Is it possible that the absorbance was too high in the phototube, again?

Answer 11: This is not an absorbance saturation issue. In the surface activity assay using the Miller unit method, cells are diluted prior to measurement to ensure OD₄₂₀ values remain below 1.0, and the dilution factor is applied in the calculation. The observed reduction in activity at 5 h induction is therefore a genuine biological effect, most likely attributable to the drop in culture pH to 5.28 (approaching the predicted pI of 5.0), which may cause LysM domain detachment from the cell wall and/or enzyme aggregation. This has been clarified in the Discussion (line 551-554).

Comment 12: The authors reported the mean +/- SD of three replicates. Where are the errors in the figures?

Answer 12: Error bars representing SD are present in both figures, but the SD values are small relative to the y-axis scale, making them difficult to see. A note clarifying this has been added to the figure captions. The figures will be regenerated with clearly visible error bars in the final revision.

Comment 13: The authors didn't see any activity after cycle 3 and they mentioned that catalytic activity was lost. Any idea why is this? Some discussion should be presented.

Answer 13: The complete loss of catalytic activity by the third cycle is attributed to the cumulative effects of: (1) mechanical shear stress during repeated centrifugation and washing between cycles, which progressively dislodges the surface-anchored enzyme; and (2) prolonged thermal stress at 50 °C, which may reduce the binding affinity of the LysM domain to peptidoglycan over successive reaction cycles. Collectively, these factors deplete the surface-anchored enzyme pool to levels insufficient for detectable product formation by the third cycle. This point has been clarify in the discussion (597-604).

Comment 14: Lines 414, As far as I understand AlphaFold3 modelling, it only predicts monomers. Why the authors predicted to be homotetramer?

Answer 14: We thank the reviewer for this valuable comment. AlphaFold3 can also be used to predict oligomeric assemblies in addition to monomeric structures. Since our enzyme exists as a homotetramer in solution, the model was generated in its homotetrameric form to better represent the biologically relevant state of the protein. Furthermore, the predicted structure was compared with the crystallographic structure of the β-galactosidase from E. coli (PDB: 1JYX), which also forms a homotetramer. This template contains IPTG bound within the active site. By superimposing our AlphaFold-predicted model with the crystal structure, we were able to identify the putative active-site region and residues potentially involved in ligand binding, including the catalytic residues. In the revised manuscript, figures 5 and 6 have been replaced with higher-resolution versions to improve clarity and overall figure quality.

Comment 15: Line 442, a magnesium ion was observed in the protein from E. coli, no in the model from AlphaFold. The way that is presented was confusing. An AlphaFold3 model only presents the model of the protein, but it doesn’t incorporate any ion, only X-ray or other lab-based technique, does that.

Answer 15: We agree that the magnesium ion was not predicted by AlphaFold3. The magnesium ion and IPTG originate from the E. coli crystal structure (PDB: 1JYX), which was used as a template for structural comparison. Structural alignment between the two proteins yielded an RMSD value of 0.518 Å over 760 aligned atoms, indicating a very high degree of structural similarity between our AlphaFold-predicted model and the experimentally determined E. coli structure. Based on this strong structural similarity and the structural superposition, we proposed the possible positions of the ligand and magnesium ion within the active site of our model.

Response to Reviewer 2

We sincerely thank both reviewers for their thorough and constructive comments. Below is our point-by-point response. Text added or revised in the manuscript is highlighted in yellow in the revised document.

Comment 1: This paper did not appear to provide the sequence of this specific Lactococcus sp. KTH0-1S β-galactosidase. Please include this information. How is the homology vs other Lactococcus species in databases?

Answer 1: We thank the reviewer for this important observation. BLAST analysis against the NCBI non-redundant protein database restricted to the genus Lactococcus revealed highest sequence identity to β-galactosidase from L. lactis (97.19%, 100% query coverage; WP_404378437.1), followed by L. kimchii (69.32%), L. taiwanensis (64.04%), L. hircilactis (58.79%), and L. termiticola (55.69%), confirming broad conservation of this GH2 enzyme within the Lactococcus genus. The manuscripts has been revised in the methods Sections 2.8 and this result has been added as suggested in Section 3.3 (line 294-300).

Comment 2: The reference source for L. sp. KTH0-1S is mentioned as 'this study'. But I believe the same strain was isolated and characterized earlier.

Answer 2: Thank you for the comment. The Table 1 has been updated to cite Reference 19 (Saelao et al., Food Control, 2018) as the original source of the strain as suggested.

Comment 3: In 2.2.3, a final 25% 1:1 lactose:sucrose was used. However, in subsequent experiments (e.g., 2.4), 12.5% solutions were used. Please explain rationale.

Answer 3: The 25% (w/v) substrate concentration in Section 2.2.3 was used for initial validation of lactosucrose synthesis capability with crude enzyme extract. Based on those results, residual substrate remained in excess at the end of the reaction, indicating the reaction was not substrate-limited. Therefore, 12.5% (w/v) was selected for whole-cell experiments in later experiments to minimize osmotic stress on intact cells and to facilitate substrate dissolution, while still providing sufficient substrate for product formation. However the first experiment with 25% substrate were conducted under different system conditions (crude enzyme from WT vs. whole cells recombinant) and were not intended for direct comparison.

Comment 4: Please check and ensure that 'In silico' should be in italics (e.g., 2.8, line 289, 483).

Answer 4: All occurrences of 'in silico' throughout the manuscript (Section 2.8 heading, 3.4 body text and discussion) have been formatted in italics in the revised document.

Comment 5: Table 4: Why pick only 2 clones since the differences are significant.

Answer 5: We thank the reviewer for this valid comment. We acknowledge that screening only two clones is a limitation of this study. The two clones were selected based on positive ONPG activity and served as representative examples to demonstrate the feasibility of the surface display system. We agree that a broader clone screening would better capture expression variability. This limitation has been discussed in the Discussion section (line 541-545) of the revised manuscript, and future work will include screening of a larger number of clones to identify variants with optimal surface display efficiency.

Comment 6: Fig 3 caption. Please define the 'identical' conditions used.

Answer 6: The Figure 3 caption has been revised to explicitly define the conditions as suggested to avoid confusion: recombinant Lactococcus sp. KTH0-1S cells at OD₆₀₀ = 20; lactose and sucrose each at 12.5% (w/v); 50 °C; 4 h incubation. (line 392-393)

Comment 7: 3:1 (and 1:3) lactose:sucrose ratios gave notable higher yields. Why not go higher (e.g., 4:1 or 1:4)?

Answer 7: This is an interesting suggestion. The molar ratios tested were constrained by substrate solubility at the concentrations used; higher concentration (4:1 or 1:4) would require one substrate at a concentration approaching its solubility limit, potentially causing precipitation and inconsistent results.

Comment 8: Secretion of USP45 tagged proteins can also be included by use of secretion propeptides and anchoring of the enzyme can be attempted with other cell anchoring domains.

Answer 8: We thank the reviewer for these valuable suggestions. As recommended, we have expanded the future perspectives section in the Discussion to include: the use of different secretion propeptides in combination with the USP45 signal peptide to enhance secretion efficiency and the exploration of alternative non-covalent cell wall anchoring domains, such as the SH3_5-based CAD4a domain, as alternatives to LysM for potentially more robust surface display on LAB have been added to the revised manuscript (line 608-613)

Attachment Submitted filename: Response to Reviewer 2.docx

# Peer review: Decision Letter 1

**DOI:** 10.1371/journal.pone.0359091.r003

**Authors:** Dirk Tischler

21 Jun 2026

Dear Dr. Watthanasakphuban,

Thank you for submitting your manuscript to PLOS ONE. After careful consideration, we feel that it has merit but does not fully meet PLOS ONE’s publication criteria as it currently stands. Therefore, we invite you to submit a revised version of the manuscript that addresses the points raised during the review process.

Please submit your revised manuscript by Aug 05 2026 11:59PM. If you will need more time than this to complete your revisions, please reply to this message or contact the journal office at plosone@plos.org. When you're ready to submit your revision, log on to https://www.editorialmanager.com/pone/ and select the 'Submissions Needing Revision' folder to locate your manuscript file.

- A letter that responds to each point raised by the academic editor and reviewer(s). You should upload this letter as a separate file labeled 'Response to Reviewers'.
- A marked-up copy of your manuscript that highlights changes made to the original version. You should upload this as a separate file labeled 'Revised Manuscript with Track Changes'.
- An unmarked version of your revised paper without tracked changes. You should upload this as a separate file labeled 'Manuscript'.

If you would like to make changes to your financial disclosure, please include your updated statement in your cover letter. Guidelines for resubmitting your figure files are available below the reviewer comments at the end of this letter.

If applicable, we recommend that you deposit your laboratory protocols in protocols.io to enhance the reproducibility of your results. Protocols.io assigns your protocol its own identifier (DOI) so that it can be cited independently in the future. For instructions see: https://journals.plos.org/plosone/s/submission-guidelines#loc-laboratory-protocols. Additionally, PLOS ONE offers an option for publishing peer-reviewed Lab Protocol articles, which describe protocols hosted on protocols.io. Read more information on sharing protocols at https://plos.org/protocols?utm_medium=editorial-email&utm_source=authorletters&utm_campaign=protocols.

As the corresponding author, your ORCID iD is verified in the submission system and will appear in the published article. PLOS supports the use of ORCID, and we encourage all coauthors to register for an ORCID iD and use it as well. Please encourage your coauthors to verify their ORCID iD within the submission system before final acceptance, as unverified ORCID iDs will not appear in the published article. *Only*  the individual author can complete the verification step; PLOS staff *cannot*  verify ORCID iDs on behalf of authors.

We look forward to receiving your revised manuscript.

Kind regards,

Dirk Tischler

Academic Editor

PLOS One

Journal Requirements:

If the reviewer comments include a recommendation to cite specific previously published works, please review and evaluate these publications to determine whether they are relevant and should be cited. There is no requirement to cite these works unless the editor has indicated otherwise.

Please review your reference list to ensure that it is complete and correct. If you have cited papers that have been retracted, please include the rationale for doing so in the manuscript text, or remove these references and replace them with relevant current references. Any changes to the reference list should be mentioned in the rebuttal letter that accompanies your revised manuscript. If you need to cite a retracted article, indicate the article’s retracted status in the References list and also include a citation and full reference for the retraction notice.

Additional Editor Comments:

As reviewer, I think either you need to proof the localization of the target protein e.g. by means of a western plot or simplify the message. All other comments have properly been addressed.

[Note: HTML markup is below. Please do not edit.]

Reviewers' comments:

Reviewer's Responses to Questions

**Comments to the Author**

Reviewer #1: (No Response)

Reviewer #2: All comments have been addressed

**********

2. Is the manuscript technically sound, and do the data support the conclusions?

Reviewer #1: Partly

Reviewer #2: Yes

**********

3. Has the statistical analysis been performed appropriately and rigorously?

Reviewer #1: N/A

Reviewer #2: Yes

**********

4. Have the authors made all data underlying the findings in their manuscript fully available?

The PLOS Data policy

Reviewer #1: Yes

Reviewer #2: Yes

**********

5. Is the manuscript presented in an intelligible fashion and written in standard English?

Reviewer #1: Yes

Reviewer #2: Yes

**********

Reviewer #1: The authors addressed all my comments, except one. I still want to see a direct proof that the enzyme is in the surface. I understand that the evidence is indirectly proofed but there is no direct evidence that the correct enzyme is in the surface. This is a major statement as the actual title of the paper reflects the ability of the study to present surface display, therefore I would like to see that proof. A simple western blot would be sufficient but it would definitely proof the presence of the target enzyme in the surface and successfully expression in that location.

In addition, the quality of the figures is not high. I see them blurry in my computer, specially the structural figures. I recommend to save the figures, I presume from Pymol, with high resolution.

Reviewer #2: (No Response)

**********

what does this mean?). If published, this will include your full peer review and any attached files.

If you choose “no”, your identity will remain anonymous but your review may still be made public.

**Do you want your identity to be public for this peer review?**  For information about this choice, including consent withdrawal, please see our Privacy Policy

Reviewer #1: No

Reviewer #2: **Yes:**  Dave Siak-Wei Ow

**********

[NOTE: If reviewer comments were submitted as an attachment file, they will be attached to this email and accessible via the submission site. Please log into your account, locate the manuscript record, and check for the action link "View Attachments". If this link does not appear, there are no attachment files.]

To ensure your figures meet our technical requirements, please review our figure guidelines: https://journals.plos.org/plosone/s/figures

You may also use PLOS’s free figure tool, NAAS, to help you prepare publication quality figures: https://journals.plos.org/plosone/s/figures#loc-tools-for-figure-preparation.

NAAS will assess whether your figures meet our technical requirements by comparing each figure against our figure specifications.

# Author comment: Author response to Decision Letter 2

**DOI:** 10.1371/journal.pone.0359091.r004

21 Aug 2026

Comment 1: “I still want to see a direct proof that the enzyme is in the surface... A simple western blot would be sufficient but it would definitely proof the presence of the target enzyme in the surface and successfully expression in that location.” (Reviewer 1)

“I think either you need to proof the localization of the target protein e.g. by means of a western plot or simplify the message.” (Editor)

Answer 1: We thank the reviewer and editor for this comment. We attempted Western blot analysis of the LiCl-extracted cell surface fraction using an anti-His antibody; however, SDS-PAGE and Western blot did not yield a clear, unambiguous band, likely due to the low abundance of the surface-displayed protein relative to the total extracted protein.

To provide direct protein-level evidence, we therefore performed dot blot analysis, a more sensitive immunodetection approach that avoids the additional dilution and transfer losses inherent to gel electrophoresis and membrane transfer. LiCl-extracted surface protein fractions from wild-type and recombinant Lactococcus sp. KTH0-1S, under both non-induced and nisin-induced conditions, were probed with anti-His antibody. A distinct immunoreactive signal was detected exclusively in the induced recombinant sample, with no signal observed in the non-induced recombinant sample or in either wild-type sample (Fig 8). This result provides direct immunological evidence that the His-tagged β-galactosidase is expressed and displayed on the cell surface, dependent on nisin induction, and complements the functional and genetic evidence presented in our previous response.

This new result and its methodology have been added to the revised manuscript (Method 2.4.3 (line 184-225), Result 3.11 (line 551-566) and Fig 8, with interpretation provided in the Discussion (line 588-596).

Attachment Submitted filename: Response_to_Reviewer_1_Round2.docx

# Editor report: Decision Letter 2

**DOI:** 10.1371/journal.pone.0359091.r005

**Authors:** Dirk Tischler

26 Aug 2026

Dear Dr. Watthanasakphuban,

Thank you for submitting your manuscript to PLOS One. After careful consideration, we feel that it has merit but does not fully meet PLOS One’s publication criteria as it currently stands. Therefore, we invite you to submit a revised version of the manuscript that addresses the points raised during the review process.

Please submit your revised manuscript by Oct 10 2026 11:59PM. If you will need more time than this to complete your revisions, please reply to this message or contact the journal office at plosone@plos.org. When you're ready to submit your revision, log on to https://www.editorialmanager.com/pone/ and select the 'Submissions Needing Revision' folder to locate your manuscript file.

- A letter that responds to each point raised by the academic editor and reviewer(s). You should upload this letter as a separate file labeled 'Response to Reviewers'.
- A marked-up copy of your manuscript that highlights changes made to the original version. You should upload this as a separate file labeled 'Revised Manuscript with Track Changes'.
- An unmarked version of your revised paper without tracked changes. You should upload this as a separate file labeled 'Manuscript'.

If you would like to make changes to your financial disclosure, please include your updated statement in your cover letter. Guidelines for resubmitting your figure files are available below the reviewer comments at the end of this letter.

If applicable, we recommend that you deposit your laboratory protocols in protocols.io to enhance the reproducibility of your results. Protocols.io assigns your protocol its own identifier (DOI) so that it can be cited independently in the future. For instructions see: https://journals.plos.org/plosone/s/submission-guidelines#loc-laboratory-protocols. Additionally, PLOS One offers an option for publishing peer-reviewed Lab Protocol articles, which describe protocols hosted on protocols.io. Read more information on sharing protocols at https://plos.org/protocols?utm_medium=editorial-email&utm_source=authorletters&utm_campaign=protocols.

As the corresponding author, your ORCID iD is verified in the submission system and will appear in the published article. PLOS supports the use of ORCID, and we encourage all coauthors to register for an ORCID iD and use it as well. Please encourage your coauthors to verify their ORCID iD within the submission system before final acceptance, as unverified ORCID iDs will not appear in the published article. *Only*  the individual author can complete the verification step; PLOS staff *cannot*  verify ORCID iDs on behalf of authors.

We look forward to receiving your revised manuscript.

Kind regards,

Dirk Tischler

Academic Editor

PLOS One

Journal Requirements:

If the reviewer comments include a recommendation to cite specific previously published works, please review and evaluate these publications to determine whether they are relevant and should be cited. There is no requirement to cite these works unless the editor has indicated otherwise.

Please review your reference list to ensure that it is complete and correct. If you have cited papers that have been retracted, please include the rationale for doing so in the manuscript text, or remove these references and replace them with relevant current references. Any changes to the reference list should be mentioned in the rebuttal letter that accompanies your revised manuscript. If you need to cite a retracted article, indicate the article’s retracted status in the References list and also include a citation and full reference for the retraction notice.

Additional Editor Comments:

Please show either evidence for the enzyme being located in the surface as stated or rephrase the statement.

[Note: HTML markup is below. Please do not edit.]

[NOTE: If reviewer comments were submitted as an attachment file, they will be attached to this email and accessible via the submission site. Please log into your account, locate the manuscript record, and check for the action link "View Attachments". If this link does not appear, there are no attachment files.]

To ensure your figures meet our technical requirements, please review our figure guidelines: https://journals.plos.org/plosone/s/figures

You may also use PLOS’s free figure tool, NAAS, to help you prepare publication quality figures: https://journals.plos.org/plosone/s/figures#loc-tools-for-figure-preparation.

NAAS will assess whether your figures meet our technical requirements by comparing each figure against our figure specifications.

# Author comment: Author response to Decision Letter 3

**DOI:** 10.1371/journal.pone.0359091.r006

8 Sep 2026

Editor Comments : Please show either evidence for the enzyme being located in the surface as stated or rephrase the statement.

Response:

We thank the Editor for this observation and have chosen to rephrase the statement throughout the manuscript. Specifically, we have replaced or removed all references to “surface display” with “expression” (or equivalent, more conservative wording such as “whole-cell biocatalyst/ whole-cell recombinant” where appropriate) throughout the manuscript. This revision has been applied consistently to:

– The manuscript title, which now “β-Galactosidase Expression on Lactococcus sp. KTH0-1S as a Whole-Cell Biocatalyst for Efficient Lactosucrose Production” (previously “Surface Display of β-Galactosidase on Lactococcus sp. KTH0-1S as a Whole-Cell Biocatalyst for Efficient Lactosucrose Production”).

– The Abstract.

Line 31 exhibited a maximum whole-cell β-galactosidase activity

Line 32 The whole-cell enzyme efficiently catalyzed lactosucrose synthesis

– The Introduction

Line 121 This work demonstrates the feasibility of employing a GRAS Lactococcus host as a safe and efficient whole-cell biocatalysis platform for cost-effective lactosucrose production

– Material and methods

Line 170 Construction of whole-cell recombinant Lactococcus sp. displaying β-galactosidase

Line 186 The whole-cell β-galactosidase activity was measured using ONPG

Line 218 To compare surface protein expression between Lactococcus sp.

Line 251-252 using whole-cell biocatalyst recombinant Lactococcus sp. with β-galactosidase (final OD₆₀₀ = 100).

- The Results sections

Line 340 Design of the β-galactosidase expression construct for whole-cell immobilized Lactococcus sp.

Line 342 to enable whole-cell biocatalysis of β-galactosidase in Lactococcus sp. KTH0-1S.

Line 364 Prediction of surface displayed protein localization

Line 382 Screening of recombinant Lactococcus sp. clones for whole-cell immobilized β-galactosidase activity

Line 383 Recombinant Lactococcus sp. KTH0-1S clones harboring the expression construct

Line 388 No detectable β-galactosidase activity was observed using whole-cell of the wild-type

Line 402 both recombinant clones exhibited a marked increase in whole-cell β-galactosidase activity compared with the corresponding non-induced controls

Line 411 Whole-cell β-galactosidase activity of recombinant Lactococcus sp. KTH0-1S clones under induced and non-induced conditions

Line 418 Effect of induction time on the activity of whole-cell β-galactosidase in recombinant Lactococcus sp.

Line 420 The effect of induction time on the activity of whole-cell β-galactosidase

Line 424 Whole-cell β-galactosidase activity increased progressively during the early induction period

Line 431 Effect of induction time on whole-cell β-galactosidase activity and culture pH

Line 439 The effect of pH on the catalytic activity of whole-cell β-galactosidase from recombinant Lactococcus sp. KTH0-1S

Line 456 lactosucrose synthesis using whole-cell β-galactosidase in Lactococcus sp. lies between pH 6.0 and 8.0.

Line 459 Effect of reaction pH on lactosucrose synthesis catalyzed by whole-cell β-galactosidase in recombinant Lactococcus sp.

Line 468-469 The effect of temperature on lactosucrose synthesis catalyzed by whole-cell β-galactosidase in recombinant Lactococcus sp.

Line 478-479 Effect of temperature and reaction time on lactosucrose production catalyzed by whole-cell β-galactosidase in recombinant Lactococcus sp. KTH0-1S.

Line 490-491 the optimal temperature for lactosucrose synthesis using whole-cell β-galactosidase under the tested conditions.

Line 494-495 The molar ratio of lactose to sucrose significantly affected lactosucrose synthesis catalyzed by whole-cell β-galactosidase in recombinant Lactococcus sp. KTH0-1S

Line 511-512 Effect of lactose:sucrose molar ratio on lactosucrose production and reusability of whole-cell β-galactosidase in recombinant Lactococcus sp. KTH0-1S.

Line 585 Confirmation of whole-cell β-galactosidase expression by dot blot

Line 591-592 These results confirm that the His-tagged β-galactosidase was successfully expressed of the recombinant Lactococcus sp. KTH0-1S

Line 597 Dot blot analysis of whole-cell β-galactosidase in Lactococcus sp. KTH0-1S.

- – The Discussion

Line 602-603 Lactococcus sp. KTH0-1S strain capable of whole-cell β-galactosidase activity

Line 613-614 this whole-cell biocatalysis approach offers several advantages, most notably the elimination of cell disruption,

Line 618-619 Nevertheless, this protein localization requires more sophisticated genetic design than intracellular expression

Line 624-625 experimental validation confirmed successful localization, with the engineered strain exhibiting β-galactosidase activity

Line 627 construction of whole-cell biocatalysis in Lactococcus sp. KTH0-1S.

Line 660-663 Future work should therefore include broader screening more recombinant colonies to better capture the full range of expression variability and to identify clones with consistently optimal whole-cell biocatalysis efficiency. Induction time was identified as another critical factor influencing whole-cell enzyme activity.

Line 673 Biochemical characterization of the whole-cell β-galactosidase demonstrated that both pH and temperature

Line 713 Despite its high initial activity, the surface-displayed whole-cell biocatalyst exhibited limited reusability

Line 737 as a whole-cell β-galactosidase platform

- Conclusion

Line 744-745 This study demonstrates the successful development of a recombinant Lactococcus sp. KTH0-1S system displaying whole-cell β-galactosidase activity for efficient lactosucrose synthesis.

Attachment Submitted filename: Response_to_Editor.docx

# Editor report: Decision Letter 3

**DOI:** 10.1371/journal.pone.0359091.r007

**Authors:** Dirk Tischler

9 Sep 2026

β-Galactosidase expression on  Lactococcus  sp. KTH0-1S as a whole-cell biocatalyst for efficient lactosucrose production

PONE-D-26-01975R3

Dear Dr. Watthanasakphuban,

We’re pleased to inform you that your manuscript has been judged scientifically suitable for publication and will be formally accepted for publication once it meets all outstanding technical requirements.

Within one week, you’ll receive an e-mail detailing the required amendments. When these have been addressed, you’ll receive a formal acceptance letter and your manuscript will be scheduled for publication.

An invoice will be generated when your article is formally accepted. Please note, if your institution has a publishing partnership with PLOS and your article meets the relevant criteria, all or part of your publication costs will be covered. Please make sure your user information is up-to-date by logging into Editorial Manager at Editorial Manager® and clicking the ‘Update My Information' link at the top of the page. For questions related to billing, please contact billing support.

If your institution or institutions have a press office, please notify them about your upcoming paper to help maximize its impact. If they’ll be preparing press materials, please inform our press team as soon as possible -- no later than 48 hours after receiving the formal acceptance. Your manuscript will remain under strict press embargo until 2 pm Eastern Time on the date of publication. For more information, please contact onepress@plos.org.

Kind regards,

Dirk Tischler

Academic Editor

PLOS One

Additional Editor Comments (optional):

Thank you for revising the manuscript and now it is sound well presented.

Reviewers' comments:

# Editor report: Acceptance letter

**DOI:** 10.1371/journal.pone.0359091.r008

**Authors:** Dirk Tischler

PONE-D-26-01975R3

PLOS One

Dear Dr. Watthanasakphuban,

I'm pleased to inform you that your manuscript has been deemed suitable for publication in PLOS One. Congratulations! Your manuscript is now being handed over to our production team.

At this stage, our production department will prepare your paper for publication. This includes ensuring the following:

* All references, tables, and figures are properly cited

* All relevant supporting information is included in the manuscript submission,

* There are no issues that prevent the paper from being properly typeset

You will receive further instructions from the production team, including instructions on how to review your proof when it is ready. Please keep in mind that we are working through a large volume of accepted articles, so please give us a few days to review your paper and let you know the next and final steps.

Lastly, if your institution or institutions have a press office, please let them know about your upcoming paper now to help maximize its impact. If they'll be preparing press materials, please inform our press team within the next 48 hours. Your manuscript will remain under strict press embargo until 2 pm Eastern Time on the date of publication. For more information, please contact onepress@plos.org.

You will receive an invoice from PLOS for your publication fee after your manuscript has reached the completed accept phase. If you receive an email requesting payment before acceptance or for any other service, this may be a phishing scheme. Learn how to identify phishing emails and protect your accounts at https://explore.plos.org/phishing.

If we can help with anything else, please email us at customercare@plos.org.

Thank you for submitting your work to PLOS One and supporting open access.

Kind regards,

PLOS One Editorial Office Staff

on behalf of

Asst. Prof. Dr. Dirk Tischler

Academic Editor

PLOS One
